# Supplementary material for: Adipose tissue biomarkers and type 2 diabetes incidence in normoglycemic participants in the MESArthritis Ancillary Study: A cohort study
Source: PLoS Med. 2021 Jul 9;18(7):e1003700. doi: 10.1371/journal.pmed.1003700 (PMC8337053; doi:10.1371/journal.pmed.1003700)
Supplement: S1 Checklist — STROBE, Strengthening the Reporting of Observational Studies in Epidemiology. (DOCX) [file pmed.1003700.s001.docx]

### **S1 Checklist. STROBE Statement**

|  | Item No | **Recommendation** | Section |
| --- | --- | --- | --- |
| **Title and Abstract** | | | |
| Title and Abstract | 1 | (a) Indicate the study’s design with a commonly used term in the title or the abstract | Title |
|  |  | (b) Provide in the abstract an informative and balanced summary of what was done and what was found | Abstract (methods and findings) |
| Introduction | | | |
| Background or Rationale | 2 | Explain the scientific background and rationale for the investigation being reported | Introduction (paragraphs 1 to 4) |
| Objectives | 3 | State specific objectives, including any prespecified hypotheses | Introduction (paragraph 5) |
| Methods | | | |
| Study Design | 4 | Present key elements of study design early in the paper | Methods (MESArthritis ancillary study) |
| Setting | 5 | Describe the setting, locations, and relevant dates, including periods of recruitment, exposure, follow-up, and data collection | Methods (MESArthritis ancillary study) |
| Participants | 6 | (a) Give the eligibility criteria, and the sources and methods of selection of participants. Describe methods of follow-up | Methods (MESArthritis ancillary study and T2D diagnosis) |
|  |  | (b) For matched studies, give matching criteria and number of exposed and unexposed | - |
| Variables | 7 | Clearly define all outcomes, exposures, predictors, potential confounders, and effect modifiers. Give diagnostic criteria, if applicable | Methods (MESArthritis ancillary study, T2D diagnosis, adipose tissue biomarkers) |
| Data Sources Measurement | 8* | For each variable of interest, give sources of data and details of methods of assessment (measurement). Describe comparability of assessment methods if there is more than one group | Methods (MESArthritis ancillary study, T2D diagnosis, adipose tissue biomarkers) |
| Bias | 9 | Describe any efforts to address potential sources of bias | Methods (statistical analysis) |
| Study Size | 10 | Explain how the study size was arrived at | Methods (statistical analysis) |
| Quantitative Variables | 11 | Explain how quantitative variables were handled in the analyses. If applicable, describe which groupings were chosen and why | Methods (statistical analysis) |
| Statistical Methods | 12 | (a) Describe all statistical methods, including those used to control for confounding | Methods (statistical analysis) |
|  |  | (b) Describe any methods used to examine subgroups and interactions | Methods (statistical analysis) |
|  |  | (c) Explain how missing data were addressed | Methods (statistical analysis) |
|  |  | (d) If applicable, explain how loss to follow-up was addressed | Methods (statistical analysis) |
|  |  | (e) Describe any sensitivity analyses | Methods (statistical analysis) |
| **Results** | | | |
| Participants | 13* | (a) Report numbers of individuals at each stage of study - e.g., numbers potentially eligible, examined for eligibility, confirmed eligible, included in the study, completing follow-up, and analyzed | Results (baseline characteristics) |
|  |  | (b) Give reasons for non-participation at each stage | - |
|  |  | (c) Consider use of a flow diagram | S1 Fig |
| Descriptive Data | 14* | (a) Give characteristics of study participants (e.g., demographic, clinical, social) and information on exposures and potential confounders | Table 1 |
|  |  | (b) Indicate number of participants with missing data for each variable of interest | S4 Fig |
|  |  | (c) Summarize follow-up time (e.g., average and total amount) | Results (baseline characteristics) |
| Outcome Data | 15* | Report numbers of outcome events or summary measures over time | Results (baseline characteristics) |
| Main Results | 16 | (a) Give unadjusted estimates and, if applicable, confounder-adjusted estimates and their precision (e.g., 95% confidence interval). Make clear which confounders were adjusted for and why they were included | Table 2 |
|  |  | (b) Report category boundaries when continuous variables were categorized | Table 2 |
|  |  | (c) If relevant, consider translating estimates of relative risk into absolute risk for a meaningful time period | - |
| Other Analyses | 17 | Report other analyses done - e.g., analyses of subgroups and interactions, and sensitivity analyses | Results (throughout) |
| **Discussion** | | | |
| Key Results | 18 | Summarize key results with reference to study objectives | Discussion (paragraph 1) |
| Limitations | 19 | Discuss limitations of the study, taking into account sources of potential bias or imprecision. Discuss both direction and magnitude of any potential bias | Discussion (paragraph 6) |
| Interpretation | 20 | Give a cautious overall interpretation of results considering objectives, limitations, multiplicity of analyses, results from similar studies, and other relevant evidence | Discussion (throughout) |
| Generalizability | 21 | Discuss the generalizability (external validity) of the study results | Discussion (paragraph 7) |
| **Other Information** | | | |
| Funding | 22 | Give the source of funding and the role of the funders for the present study and, if applicable, for the original study on which the present article is based | Funding |

* Give information separately for exposed and unexposed groups.
