# Supplementary material for: Adipose tissue biomarkers and type 2 diabetes incidence in normoglycemic participants in the MESArthritis Ancillary Study: A cohort study
Source: PLoS Med. 2021 Jul 9;18(7):e1003700. doi: 10.1371/journal.pmed.1003700 (PMC8337053; doi:10.1371/journal.pmed.1003700)
Supplement: S2 Table — SD, standard deviation. (DOCX) [file pmed.1003700.s003.docx]

### **S2 Table. Statistical Analyses**

| **Analysis** | **Exposure** | **Outcome** | **Planned Approach** | **Operationalized Approach** | **Comment** |
| --- | --- | --- | --- | --- | --- |
| Descriptive Analyses | - | - | Adipose tissue biomarkers will be compared using the Kruskal–Wallis one-way analysis of variance and violin plots among the participants with diabetes, prediabetes, and normoglycemia at baseline. | Participants with diabetes at baseline were excluded from this study, and adipose tissue biomarkers were compared among the participants with and without diabetes incidence over the follow-up using descriptive statistics. | Participants with diabetes were excluded before this study, and hence, their baseline characteristics are not reported. |
| Correlation Studies | - | - | - | The correlations between adipose tissue biomarkers with diabetes risk factors, homeostatic model assessment – insulin resistance, body mass index, and waist circumference were analyzed using the Pearson correlation analysis and illustrated using a symmetric correlation matrix. | The correlation studies were included to provide a better understanding of the correlation between adipose tissue biomarkers and diabetes risk factors. |
| Generalized Additive Modeling | Adipose Tissue Biomarkers  (per 1-SD increment) | Diabetes Incidence | - | The generalized additive models with integrated smoothness estimation was used to assess and illustrate the linearity of the associations between adipose tissue biomarkers and diabetes incidence. | The generalized additive modeling was included before Cox proportional hazard modeling to assess the linearity of the associations between adipose tissue biomarkers and diabetes incidence (data-driven). |
| Cox Proportional Hazard Modeling | Adipose Tissue Biomarkers  (per 1-SD increment) | Diabetes Incidence | Using the complete-case approach, associations between adipose tissue biomarkers (log-transformed) with diabetes incidence will be studied using the Cox proportional hazard models adjusted for relevant confounders. | After multiply-imputing missing data points, associations between adipose tissue biomarkers (per 1-SD increment) with diabetes incidence was studied using the Cox proportional hazard models adjusted for relevant confounders, separately for participants with prediabetes and normoglycemia at baseline. | Per the peer-reviewer’s comments, the missing data points were multiply-imputed using the Multivariate Imputation by Chained Equations (MICE) and the predictive mean matching method. Moreover, the biomarkers were not log-transformed, and instead, the associations between adipose tissue biomarkers and diabetes incidence were reported per 1-SD increment in the biomarker (peer-reviewer’s comments). |
| Stratified Analyses | Adipose Tissue Biomarkers  (per 1-SD increment) | Diabetes Incidence | - | Stratified Cox proportional hazard modeling in the levels of traditional risk factors of diabetes, homeostatic model assessment – insulin resistance, body mass index, and waist circumference were conducted. | The stratified analyses were included to assess the association between adipose tissue biomarkers and diabetes incidence in the levels of traditional risk factors of diabetes, homeostatic model assessment – insulin resistance, body mass index, and waist circumference (data-driven). |

SD: Standard Deviation
