# Supplementary material for: Adipose tissue biomarkers and type 2 diabetes incidence in normoglycemic participants in the MESArthritis Ancillary Study: A cohort study
Source: PLoS Med. 2021 Jul 9;18(7):e1003700. doi: 10.1371/journal.pmed.1003700 (PMC8337053; doi:10.1371/journal.pmed.1003700)
Supplement: S3 Table — Model 0: unadjusted. Model 1: adjusted for categorical age, sex, race/ethnicity, smoking status, alcohol drinking status, physical activity, TG, HDL cholesterol, and hypertension. Reported p-values were corrected for multiple comparisons. BMI, body mass index; CI, confidence interval; HDL, high-density lipoprotein; HOMA-IR, homeostatic model assessment–insulin resistance; HR, hazard ratio; HU, Hounsfield unit; PM, pectoralis muscle; PY, person-year; SD, standard deviation; T2D, type 2 diabetes; TG, triglyceride; Waist C., waist circumference. (DOCX) [file pmed.1003700.s004.docx]

### **S3 Table. Associations of PM Density and Type 2 Diabetes Incidence**

|  | **Index** | | | | | | | | | **p-value for Trend** | | **HR (95% CI), p-value**  per 1-SD increment |
| --- | --- | --- | --- | --- | --- | --- | --- | --- | --- | --- | --- | --- |
|  | Quartile 1 | | Quartile 2 | | Quartile 3 | | Quartile 4 | | |  |  |  |
| **PM Density** | | | | | | | | | | |  | |
| Mean (HU/cm^2^) | | 10.4 | | 20.9 | | 27.8 | | 36.7 | - | | - | |
| Incident Cases | | 30 | | 25 | | 20 | | 28 | - | | - | |
| Incidence Rate (per 1,000 PYs) | | 11.1 | | 9.2 | | 7.3 | | 10.3 | - | | - | |
| HR (95% CI) | | | | | | | | | | |  | |
| Model 0 | | 1 (reference) | | 0.84 (0.49 - 1.44) | | 0.66 (0.37 - 1.18) | | 0.93 (0.55 - 1.57) | 0.884 | | 0.94 (0.78 - 1.15), 0.812 | |
| Model 1 | | 1 (reference) | | 0.82 (0.47 - 1.42) | | 0.58 (0.31 - 1.07) | | 0.82 (0.42 - 1.59) | 0.565 | | 0.88 (0.69 - 1.12), 0.548 | |
| Model 1 + HOMA-IR | | 1 (reference) | | 0.87 (0.50 - 1.51) | | 0.68 (0.37 - 1.27) | | 0.96 (0.49 - 1.88) | 0.909 | | 0.93 (0.73 - 1.18), 0.812 | |
| Model 1 + BMI and Waist C. | | 1 (reference) | | 0.93 (0.53 - 1.62) | | 0.70 (0.36 - 1.35) | | 1.02 (0.50 - 2.08) | 0.910 | | 0.97 (0.75 - 1.27), 0.910 | |

Model 0: Unadjusted

Model 1: Adjusted for categorical age, sex, race/ethnicity, smoking status, alcohol drinking status, physical activity, TG, HDL cholesterol, and hypertension

Reported p-values were corrected for multiple comparisons.

BMI: Body Mass Index; CI: Confidence Interval; HDL: High-density Lipoprotein; HOMA-IR: Homeostatic Model Assessment – Insulin Resistance; HR: Hazard Ratio; HU: Hounsfield Unit; PM: Pectoralis Muscles; PYs: Person-Years; SD: Standard Deviation; TG: Triglyceride; Waist C.: Waist Circumference
