# Supplementary material for: Adipose tissue biomarkers and type 2 diabetes incidence in normoglycemic participants in the MESArthritis Ancillary Study: A cohort study
Source: PLoS Med. 2021 Jul 9;18(7):e1003700. doi: 10.1371/journal.pmed.1003700 (PMC8337053; doi:10.1371/journal.pmed.1003700)
Supplement: S4 Table — Model 0: unadjusted. Model 1: adjusted for categorical age, sex, race/ethnicity, smoking status, alcohol drinking status, physical activity, TG, HDL cholesterol, and hypertension. In this sensitivity analysis, self-reported physician-diagnosed T2D (the second criterion) was confirmed with the use of insulin or oral hypoglycemic agents or FPG ≥126 mg/dL in the follow-up exam. Participants with self-reported T2D but missing information on the use of insulin or oral hypoglycemic agents or FPG in the follow-up exam (n = 17) were excluded. Reported p-values were corrected for multiple comparisons. BMI, body mass index; CI, confidence interval; FPG, fasting plasma glucose; HDL, high-density lipoprotein; HOMA-IR, homeostatic model assessment–insulin resistance; HR, hazard ratio; HU, Hounsfield unit; IMAT, intermuscular adipose tissue; PM, pectoralis muscle; PY, person-year; SAT, subcutaneous adipose tissue; SD, standard deviation; T2D, type 2 diabetes; TG, triglyceride; Waist C., waist circumference. (DOCX) [file pmed.1003700.s005.docx]

### **S4 Table. Associations of Adipose Tissue Biomarkers and Type 2 Diabetes Incidence (sensitivity analysis)**

|  | **Index** | | | | **p-value for Trend** | **HR (95% CI), p-value**  per 1-SD increment |
| --- | --- | --- | --- | --- | --- | --- |
|  | Quartile 1 | Quartile 2 | Quartile 3 | Quartile 4 |  |  |
| **IMAT Index** | | | | | | |
| Mean (cm^2^/m^2^) | 0.1 | 0.2 | 0.3 | 0.7 | - | - |
| Incident Cases | 11 | 15 | 16 | 32 | - | - |
| Incidence Rate (per 1,000 PYs) | 4.1 | 5.4 | 5.9 | 11.8 | - | - |
| HR (95% CI) | | | | | | |
| Model 0 | 1 (reference) | 1.36 (0.61 - 2.99) | 1.42 (0.65 - 3.11) | 2.94 (1.47 - 5.91) | 0.008 | 1.30 (1.19 - 1.41), <0.001 |
| Model 1 | 1 (reference) | 1.14 (0.49 - 2.61) | 1.07 (0.47 - 2.45) | 1.93 (0.90 - 4.13) | 0.134 | 1.32 (1.19 - 1.48), <0.001 |
| Model 1 + HOMA-IR | 1 (reference) | 1.11 (0.49 - 2.55) | 0.91 (0.39 - 2.13) | 1.67 (0.77 - 3.59) | 0.262 | 1.31 (1.17 - 1.47), <0.001 |
| Model 1 + BMI and Waist C. | 1 (reference) | 0.91 (0.39 - 2.12) | 0.77 (0.32 - 1.83) | 1.18 (0.51 - 2.74) | 0.721 | 1.26 (1.11 - 1.44), 0.006 |
| **SAT Index** | | | | | | |
| Mean (cm^2^/m^2^) | 7.6 | 13.1 | 20.3 | 34.9 | - | - |
| Incident Cases | 12 | 17 | 22 | 23 | - | - |
| Incidence Rate (per 1,000 PYs) | 4.6 | 6.2 | 7.9 | 8.4 | - | - |
| HR (95% CI) | | | | | | |
| Model 0 | 1 (reference) | 1.32 (0.62 - 2.81) | 1.65 (0.81 - 3.39) | 1.73 (0.85 - 3.53) | 0.204 | 1.23 (1.00 - 1.50), 0.125 |
| Model 1 | 1 (reference) | 1.37 (0.63 - 2.98) | 2.24 (1.01 - 4.95) | 3.32 (1.28 - 8.62) | 0.034 | 1.49 (1.16 - 1.90), 0.008 |
| Model 1 + HOMA-IR | 1 (reference) | 1.22 (0.56 - 2.67) | 1.70 (0.74 - 3.90) | 2.31 (0.86 - 6.21) | 0.179 | 1.35 (1.03 - 1.78), 0.090 |
| Model 1 + BMI and Waist C. | 1 (reference) | 1.11 (0.50 - 2.47) | 1.45 (0.59 - 3.56) | 1.59 (0.49 - 5.18) | 0.570 | 1.19 (0.83 - 1.70), 0.536 |
| **PM Density** | | | | | | |
| Mean (HU/cm^2^) | 10.5 | 21.0 | 27.8 | 36.8 | - | - |
| Incident Cases | 22 | 16 | 15 | 21 | - | - |
| Incidence Rate (per 1,000 PYs) | 8.1 | 5.9 | 5.5 | 7.7 | - | - |
| HR (95% CI) | | | | | | |
| Model 0 | 1 (reference) | 0.74 (0.39 - 1.43) | 0.69 (0.35 - 1.35) | 0.97 (0.53 - 1.78) | 0.931 | 1.00 (0.79 - 1.26), >0.999 |
| Model 1 | 1 (reference) | 0.75 (0.38 - 1.45) | 0.53 (0.26 - 1.11) | 0.76 (0.35 - 1.66) | 0.536 | 0.90 (0.68 - 1.21), 0.693 |
| Model 1 + HOMA-IR | 1 (reference) | 0.78 (0.40 - 1.52) | 0.66 (0.32 - 1.38) | 0.93 (0.42 - 2.04) | 0.840 | 0.97 (0.72 - 1.29), 0.931 |
| Model 1 + BMI and Waist C. | 1 (reference) | 0.91 (0.46 - 1.81) | 0.75 (0.34 - 1.64) | 1.14 (0.49 - 2.63) | 0.931 | 1.10 (0.80 - 1.52), 0.719 |

Model 0: Unadjusted

Model 1: Adjusted for categorical age, sex, race/ethnicity, smoking status, alcohol drinking status, physical activity, TG, HDL cholesterol, and hypertension

In this sensitivity analysis, self-reported physician-diagnosed type 2 diabetes (the second criterion) was confirmed with the use of insulin or oral hypoglycemic agents or fasting plasma glucose ≥126 mg/dL in the follow-up exam. Participants with self-reported type 2 diabetes but missing information on the use of insulin or oral hypoglycemic agents or fasting plasma glucose in the follow-up exam (n = 17) were excluded. Reported p-values were corrected for multiple comparisons.

BMI: Body Mass Index; CI: Confidence Interval; HDL: High-density Lipoprotein; HOMA-IR: Homeostatic Model Assessment – Insulin Resistance; HR: Hazard Ratio; HU: Hounsfield Unit; IMAT: Intermuscular Adipose Tissue; PM: Pectoralis Muscles; PYs: Person-Years; SAT: Subcutaneous Adipose Tissue; SD: Standard Deviation; TG: Triglyceride; Waist C.: Waist Circumference
