# Supplementary material for: Adipose tissue biomarkers and type 2 diabetes incidence in normoglycemic participants in the MESArthritis Ancillary Study: A cohort study
Source: PLoS Med. 2021 Jul 9;18(7):e1003700. doi: 10.1371/journal.pmed.1003700 (PMC8337053; doi:10.1371/journal.pmed.1003700)
Supplement: S5 Table — *Models did not meet the proportional hazard assumption. Model 0: unadjusted. Model 1: adjusted for categorical age, sex, race/ethnicity, smoking status, alcohol drinking status, physical activity, TG, HDL cholesterol, and hypertension. In this supplementary analysis, Cox proportional hazard models were used to study the associations between adipose tissue biomarkers and T2D incidence in participants with prediabetes at baseline. Reported p-values were corrected for multiple comparisons. BMI, body mass index; CI, confidence interval; HDL, high-density lipoprotein; HOMA-IR, homeostatic model assessment–insulin resistance; HR, hazard ratio; HU, Hounsfield unit; IMAT, intermuscular adipose tissue; PM, pectoralis muscle; PY, person-year; SAT, subcutaneous adipose tissue; SD, standard deviation; T2D, type 2 diabetes; TG, triglyceride; Waist C., waist circumference. (DOCX) [file pmed.1003700.s006.docx]

### **S5 Table. Associations of Adipose Tissue Biomarkers and Type 2 Diabetes Incidence in Participants with Prediabetes (supplementary analysis)**

|  | **Index** | | | | **p-value for Trend** | **HR (95% CI), p-value**  per 1-SD increment |
| --- | --- | --- | --- | --- | --- | --- |
|  | Quartile 1 | Quartile 2 | Quartile 3 | Quartile 4 |  |  |
| **IMAT Index** | | | | | | |
| Mean (cm^2^/m^2^) | 0.1 | 0.2 | 0.4 | 1.0 | - | - |
| Incident Cases | 29 | 46 | 40 | 49 | - | - |
| Incidence Rate (per 1,000 PYs) | 31.3 | 53.4 | 46.8 | 59.0 | - | - |
| HR (95% CI) | | | | | | |
| Model 0 | 1 (reference) | 1.72 (1.07 - 2.75) | 1.53 (0.94 - 2.47) | 1.95 (1.23 - 3.10) | 0.192 | 1.01 (0.88 - 1.16), 0.998 |
| Model 1 | 1 (reference) | 1.70 (1.04 - 2.77) | 1.44 (0.86 - 2.40) | 1.91 (1.14 - 3.19) | 0.331* | 1.00 (0.85 - 1.16), 0.998 |
| Model 1 + HOMA-IR | 1 (reference) | 1.57 (0.96 - 2.57) | 1.29 (0.76 - 2.17) | 1.65 (0.98 - 2.80) | 0.456* | 0.98 (0.82 - 1.16), 0.991 |
| Model 1 + BMI and Waist C. | 1 (reference) | 1.58 (0.96 - 2.59) | 1.28 (0.75 - 2.18) | 1.56 (0.88 - 2.75) | 0.494* | 0.90 (0.74 - 1.09), 0.494 |
| **SAT Index** | | | | | | |
| Mean (cm^2^/m^2^) | 8.5 | 13.5 | 19.4 | 35.4 | - | - |
| Incident Cases | 37 | 42 | 34 | 51 | - | - |
| Incidence Rate (per 1,000 PYs) | 41.0 | 49.5 | 38.8 | 60.4 | - | - |
| HR (95% CI) | | | | | | |
| Model 0 | 1 (reference) | 1.21 (0.77 - 1.89) | 0.95 (0.59 - 1.52) | 1.42 (0.92 - 2.17) | 0.490 | 1.20 (1.03 - 1.39), 0.192 |
| Model 1 | 1 (reference) | 1.12 (0.71 - 1.78) | 0.86 (0.51 - 1.46) | 1.21 (0.65 - 2.26) | 0.991 | 1.25 (0.99 - 1.57), 0.331 |
| Model 1 + HOMA-IR | 1 (reference) | 1.04 (0.65 - 1.66) | 0.67 (0.39 - 1.16) | 0.90 (0.47 - 1.71) | 0.702 | 1.16 (0.91 - 1.47), 0.490 |
| Model 1 + BMI and Waist C. | 1 (reference) | 0.95 (0.59 - 1.53) | 0.62 (0.35 - 1.09) | 0.62 (0.29 - 1.35) | 0.436 | 1.04 (0.75 - 1.43), 0.991 |
| **PM Density** | | | | | | |
| Mean (HU/cm^2^) | 10.1 | 20.9 | 28.5 | 37.0 | - | - |
| Incident Cases | 39 | 44 | 41 | 40 | - | - |
| Incidence Rate (per 1,000 PYs) | 47.0 | 50.1 | 45.6 | 46.4 | - | - |
| HR (95% CI) | | | | | | |
| Model 0 | 1 (reference) | 1.07 (0.70 - 1.66) | 1.00 (0.64 - 1.56) | 1.03 (0.66 - 1.60) | 0.998 | 1.00 (0.86 - 1.16), 0.998 |
| Model 1 | 1 (reference) | 1.24 (0.78 - 1.95) | 1.13 (0.68 - 1.88) | 1.23 (0.69 - 2.18) | 0.810* | 1.07 (0.87 - 1.31), 0.765 |
| Model 1 + HOMA-IR | 1 (reference) | 1.29 (0.82 - 2.05) | 1.24 (0.74 - 2.06) | 1.47 (0.82 - 2.65) | 0.490* | 1.14 (0.92 - 1.41), 0.490 |
| Model 1 + BMI and Waist C. | 1 (reference) | 1.46 (0.91 - 2.33) | 1.43 (0.84 - 2.43) | 1.65 (0.91 - 3.01) | 0.453* | 1.22 (0.98 - 1.51), 0.331 |

* Models did not meet the proportional hazard assumption

Model 0: Unadjusted

Model 1: Adjusted for categorical age, sex, race/ethnicity, smoking status, alcohol drinking status, physical activity, TG, HDL cholesterol, and hypertension

In this supplementary analysis, Cox proportional hazard models were used to study the associations between adipose tissue biomarkers and type 2 diabetes incidence in participants with prediabetes at baseline. Reported p-values were corrected for multiple comparisons.

BMI: Body Mass Index; CI: Confidence Interval; HDL: High-density Lipoprotein; HOMA-IR: Homeostatic Model Assessment – Insulin Resistance; HR: Hazard Ratio; HU: Hounsfield Unit; IMAT: Intermuscular Adipose Tissue; PM: Pectoralis Muscles; PYs: Person-Years; SAT: Subcutaneous Adipose Tissue; SD: Standard Deviation; TG: Triglyceride; Waist C.: Waist Circumference
