# Supplementary figures and images for: Adipose tissue biomarkers and type 2 diabetes incidence in normoglycemic participants in the MESArthritis Ancillary Study: A cohort study
Source: PLoS Med. 2021 Jul 9;18(7):e1003700. doi: 10.1371/journal.pmed.1003700 (PMC8337053; doi:10.1371/journal.pmed.1003700)

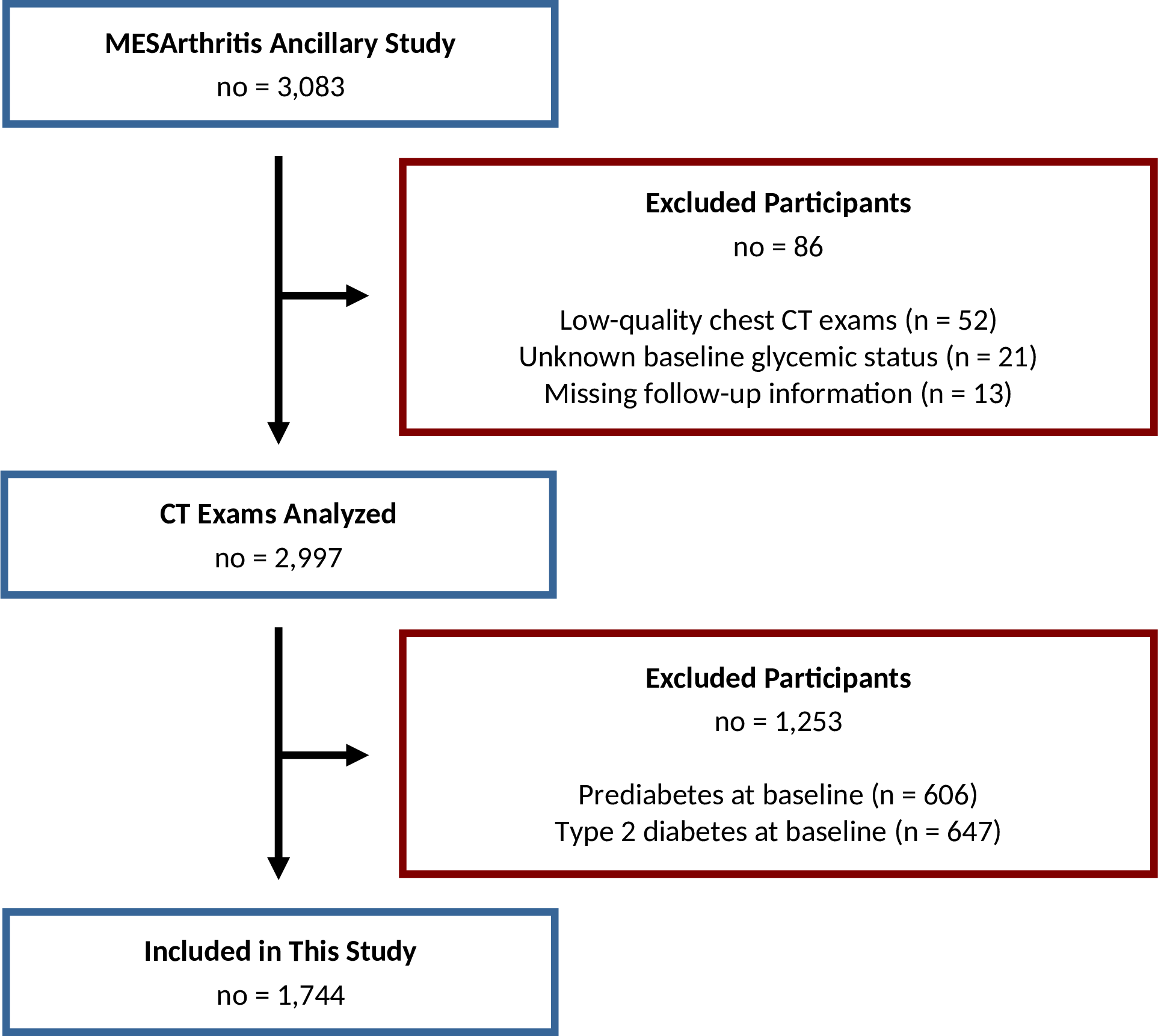

Supplement: S1 Fig — CT, computed tomography. (TIF) [file pmed.1003700.s007.tif]

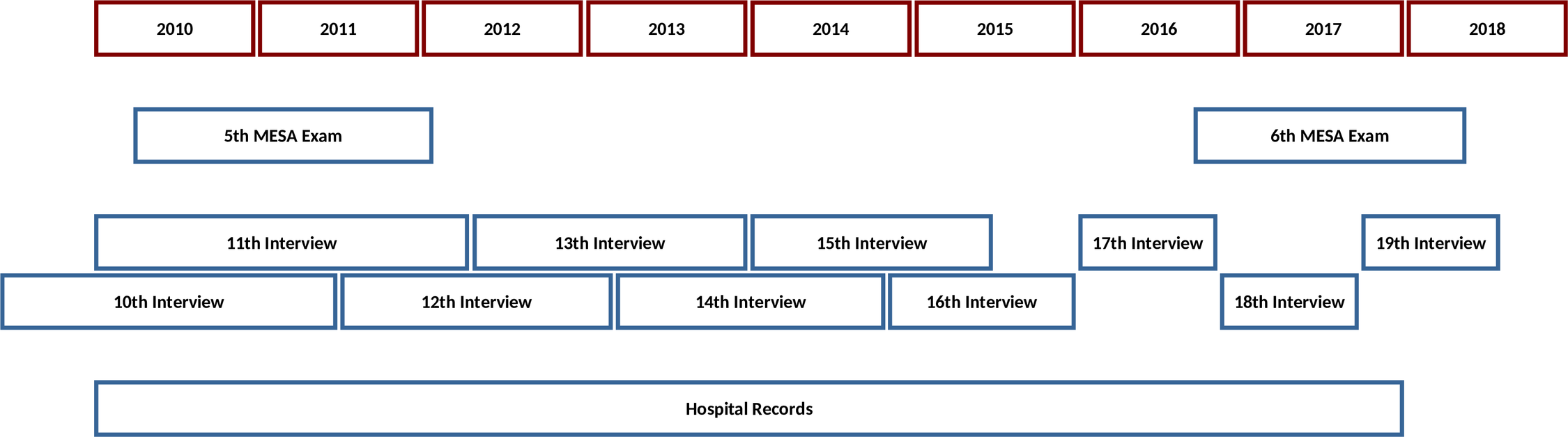

Supplement: S2 Fig — MESA, Multi-Ethnic Study of Atherosclerosis. (TIF) [file pmed.1003700.s008.tif]

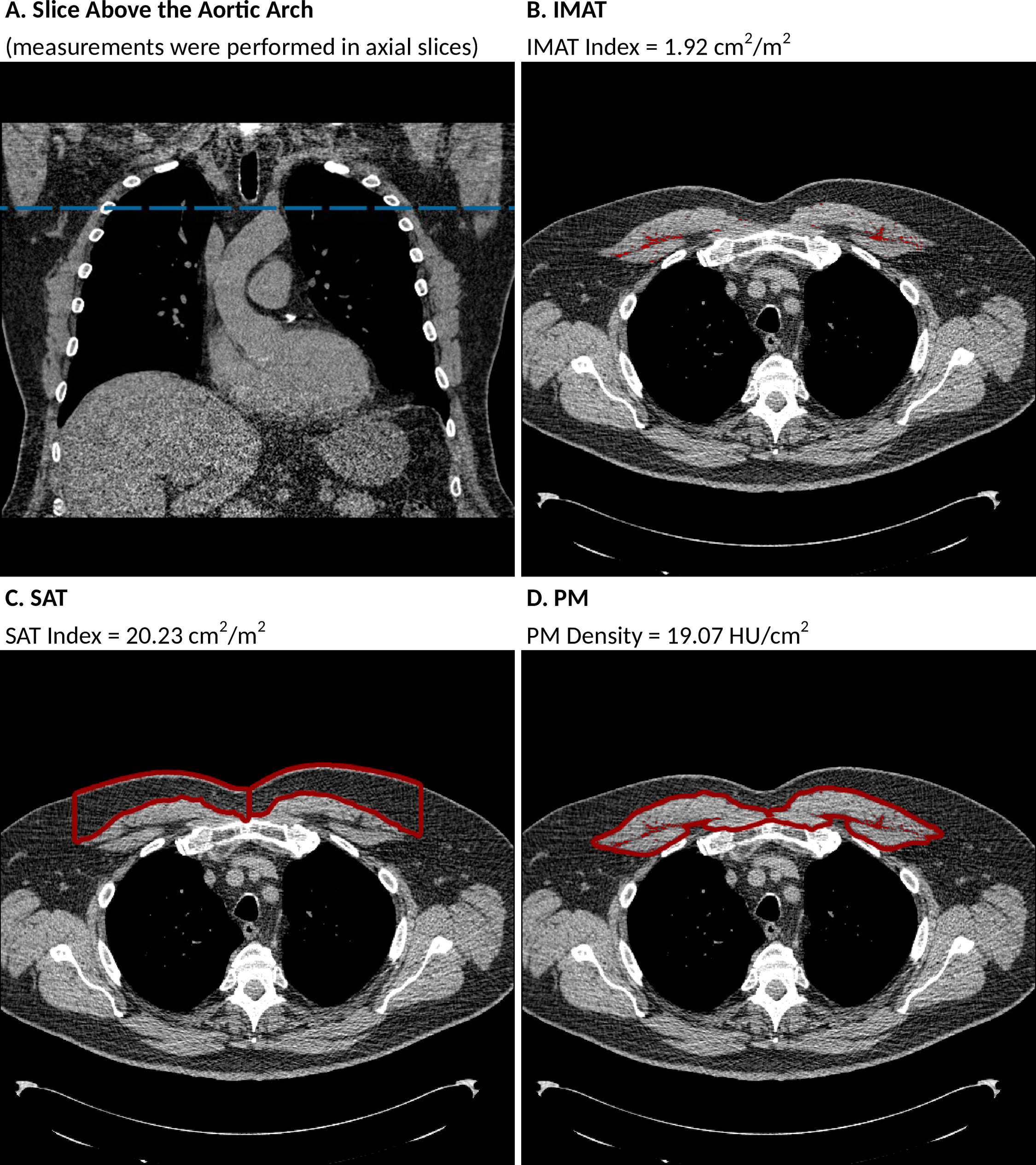

Supplement: S3 Fig — The chest CT exam of a 69-year-old normoglycemic male participant is shown here. (A) The coronal reconstruction of the CT exam is shown. The blue dashed line indicates the slice above the superior aspect of the aortic arch. (B) The area within the PM with attenuation below an individualized threshold (−89 HU in this participant) was measured as the IMAT (red). (C) The area between the PM and skin surface was measured as the SAT (red). (D) The density of PM (red) was measured as the mean HU of the pixels in the PM (after removing the pixels in the IMAT). CT, computed tomography; HU, Hounsfield unit; IMAT, intermuscular adipose tissue; PM, pectoralis muscle; SAT, subcutaneous adipose tissue. (TIF) [file pmed.1003700.s009.tif]

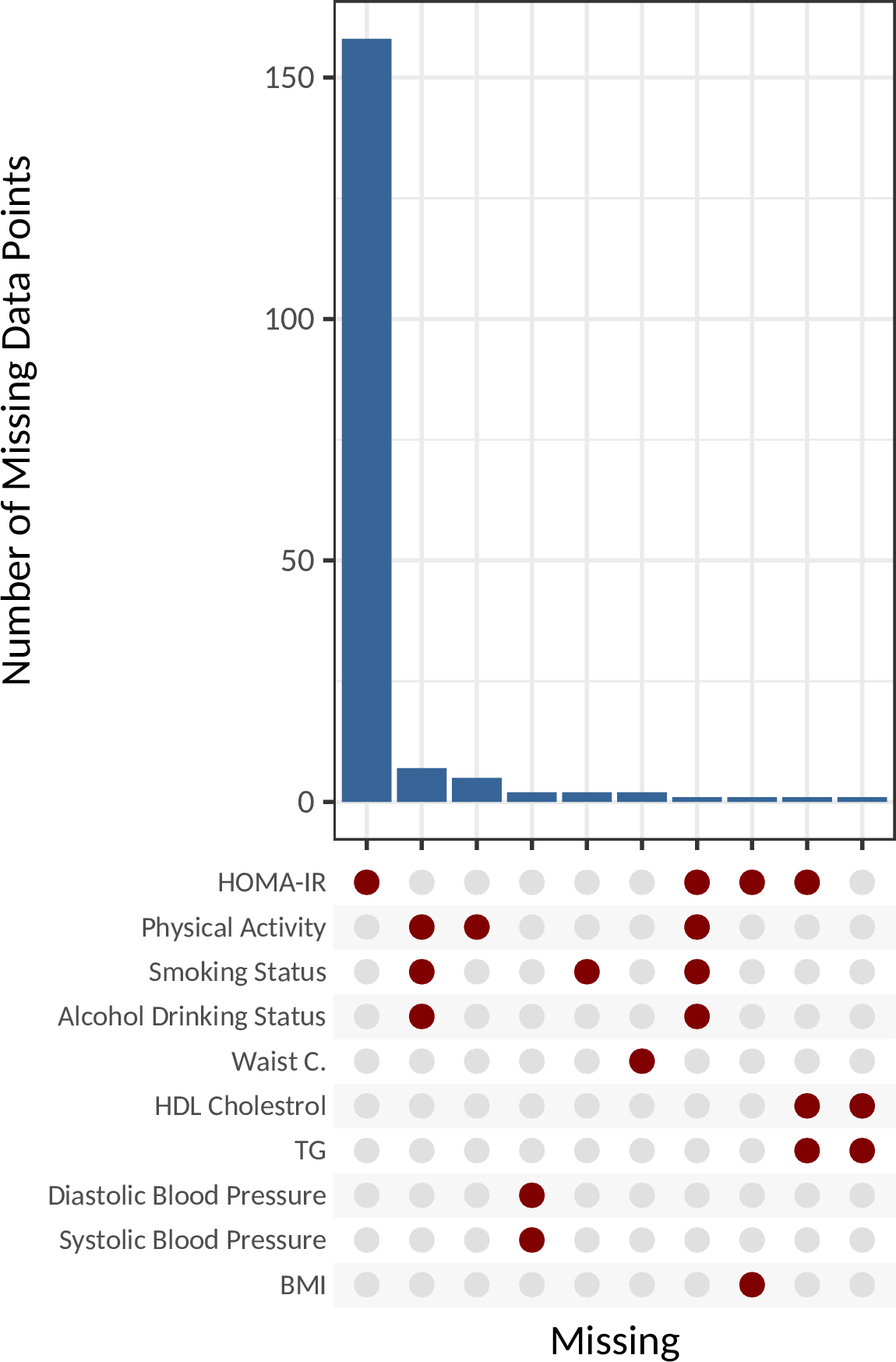

Supplement: S4 Fig — In the dataset, there were missing data points in the HOMA-IR (161 [9.2%] data points), physical activity (13 [0.7%] data points), smoking status (10 [0.6%] data points), alcohol drinking status (8 [0.5%] data points), TG, HDL cholesterol, systolic and diastolic blood pressures, BMI, and waist circumference (2 [0.1%] data points in each). In this figure, the blue bars show number of participants with missing data points in the covariates marked with red circles below each bar. BMI, body mass index; HDL, high-density lipoprotein cholesterol; HOMA-IR, homeostatic model assessment–insulin resistance; TG, triglyceride; Waist C., waist circumference. (TIF) [file pmed.1003700.s010.tif]

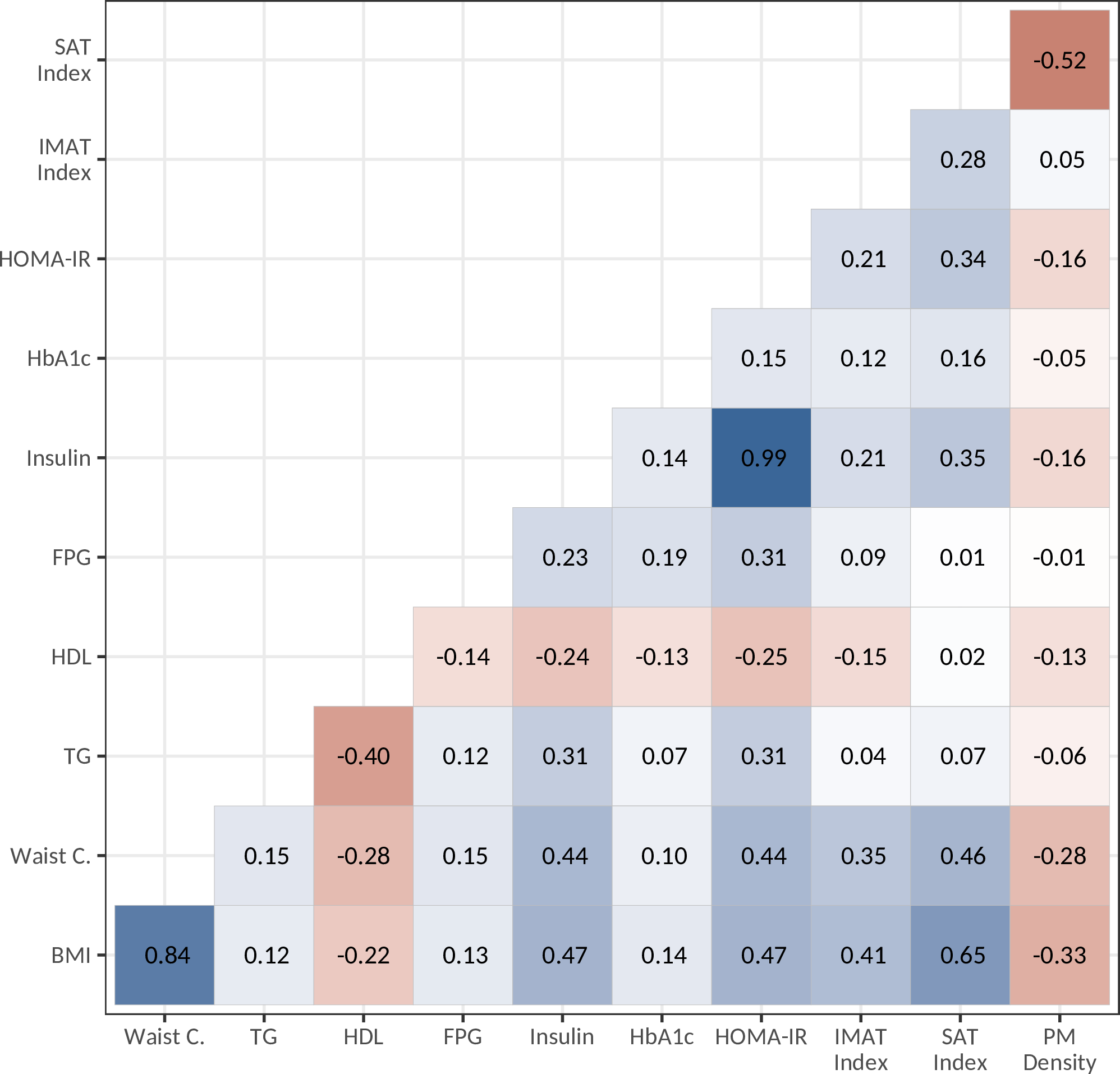

Supplement: S5 Fig — The blue color is used to display the positive correlations, and the red color is used for negative correlations. The attenuation of the color is proportional to the estimated Pearson correlation coefficients (numbers in the boxes). BMI, body mass index; FPG, fasting plasma glucose; HDL, high-density lipoprotein; HOMA-IR, homeostatic model assessment–insulin resistance; IMAT, intermuscular adipose tissue; PM, pectoralis muscle; SAT, subcutaneous adipose tissue; T2D, type 2 diabetes; TG, triglyceride; Waist C., waist circumference. (TIF) [file pmed.1003700.s011.tif]

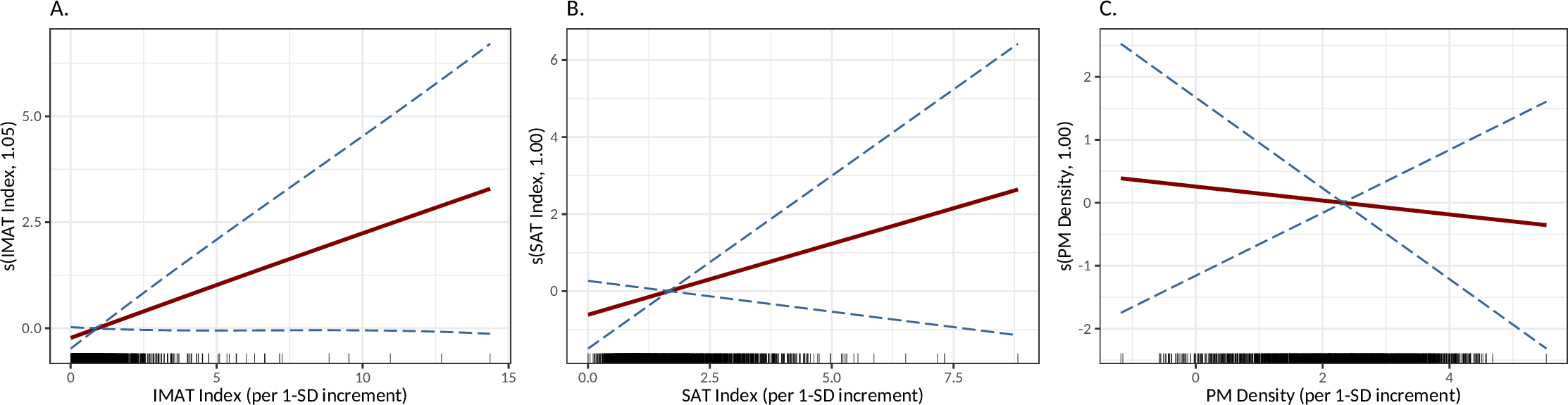

Supplement: S6 Fig — Smooth function estimates (red lines) obtained from fitting a generalized additive Cox proportional hazard models with integrated smoothness estimation on the dataset, with estimated 95% CI (blue lines) is shown. The results are reported on the scale of the adipose tissue biomarkers (per 1-SD increment), and the models were adjusted for covariates in Model 1 (categorical age, sex, race/ethnicity, smoking status, alcohol drinking status, physical activity, TG, HDL cholesterol, and hypertension). The numbers in brackets in the captions are the estimated degrees of freedom of the smooth curves. The rug marks along the x-axis indicate the adipose tissue biomarkers values. CI, confidence interval; HDL, high-density lipoprotein; HOMA-IR, homeostatic model assessment–insulin resistance; IMAT, intermuscular adipose tissue; PM, pectoralis muscle; SAT, subcutaneous adipose tissue; SD, standard deviation; T2D, type 2 diabetes; TG, triglyceride. (TIF) [file pmed.1003700.s012.tif]

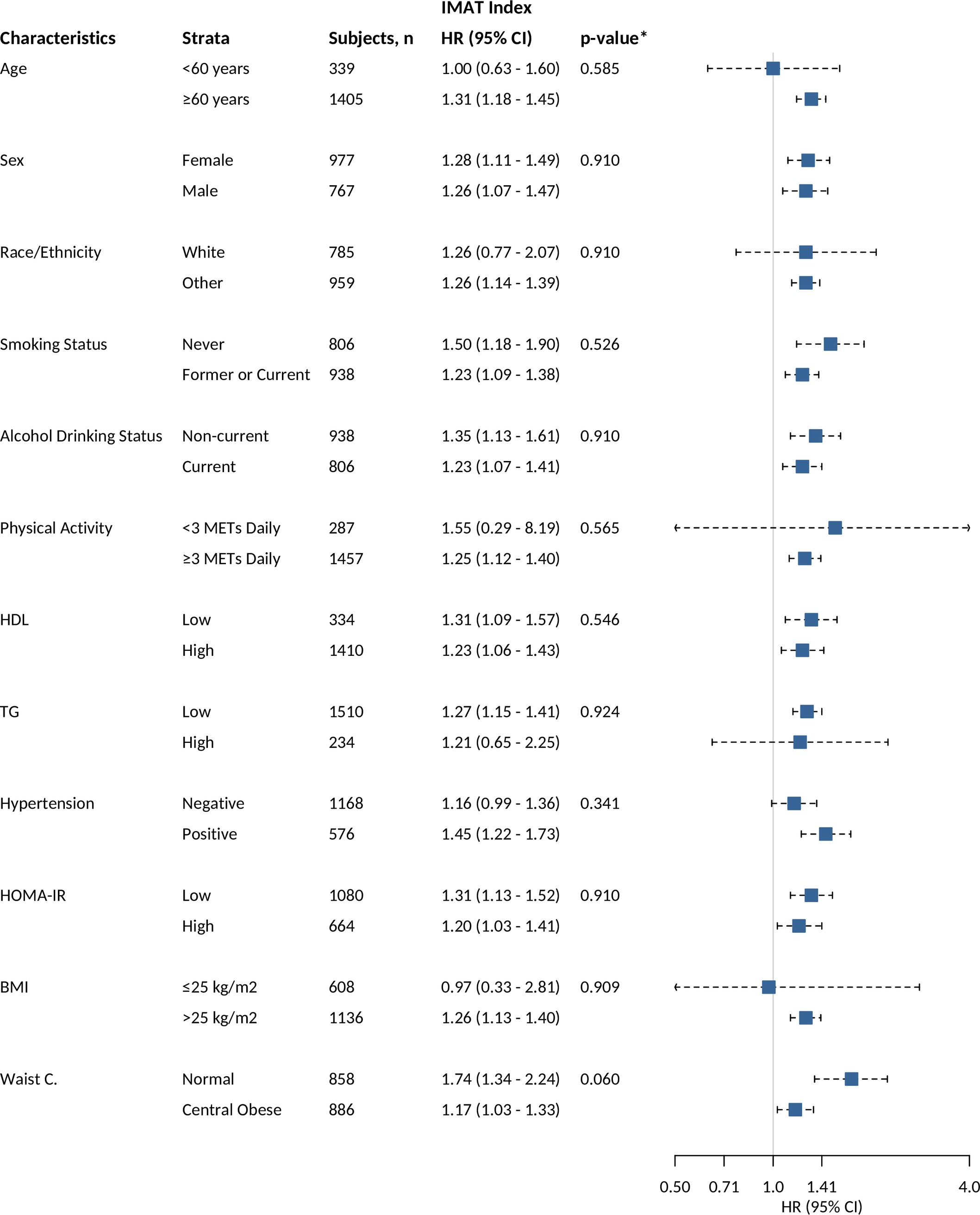

Supplement: S7 Fig — *p-value for interaction. Models were adjusted for covariates in Model 1 (categorical age, sex, race/ethnicity, smoking status, alcohol drinking status, physical activity, TG, HDL cholesterol, and hypertension), except for the stratification variable. Reported p-values were corrected for multiple comparisons. BMI, body mass index; CI, confidence interval; HDL, high-density lipoprotein; HOMA-IR, homeostatic model assessment–insulin resistance; HR, hazard ratio; IMAT, intermuscular adipose tissue; T2D, type 2 diabetes; TG, triglyceride; Waist C., waist circumference. (TIF) [file pmed.1003700.s013.tif]

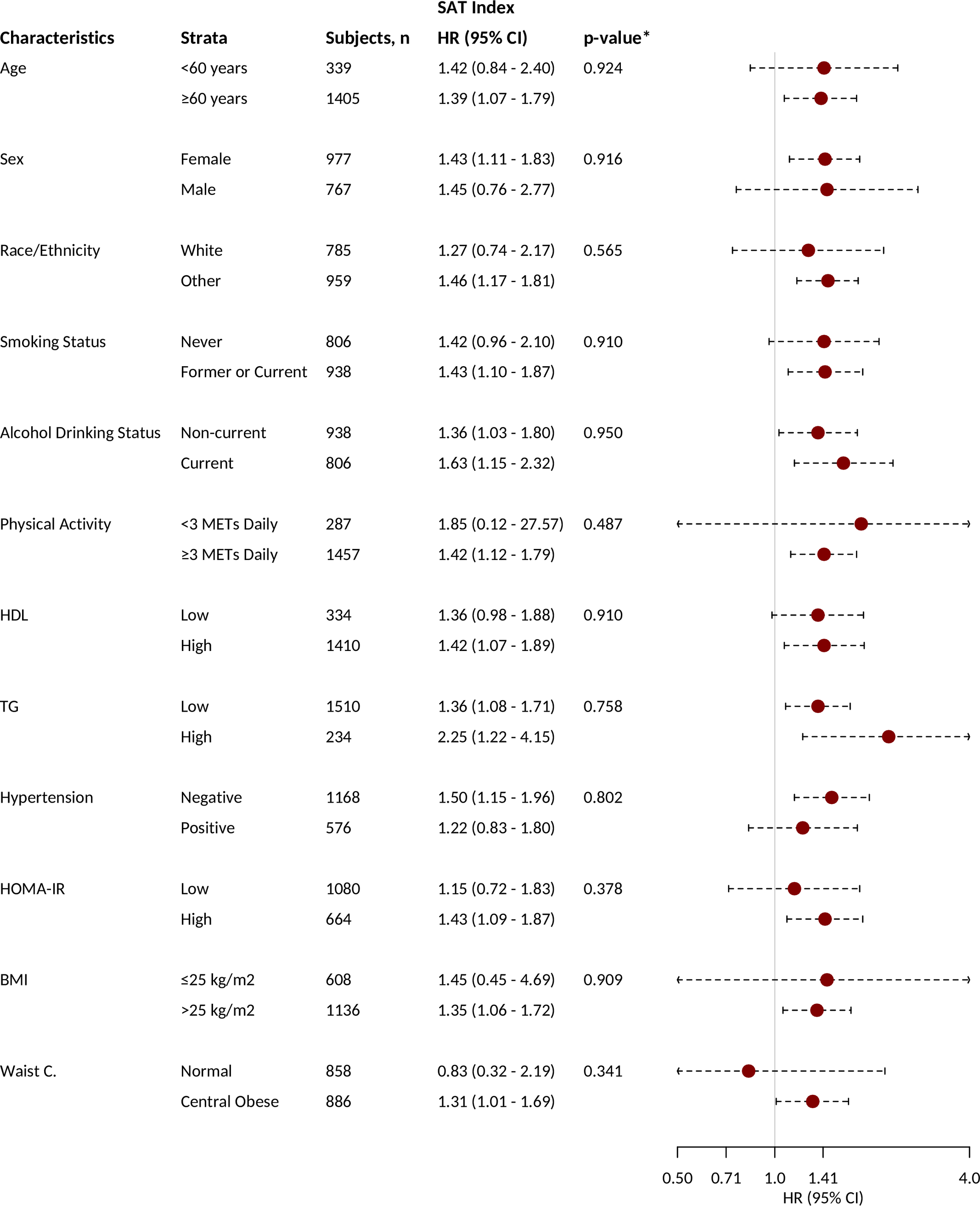

Supplement: S8 Fig — *p-value for interaction. Models were adjusted for covariates in Model 1 (categorical age, sex, race/ethnicity, smoking status, alcohol drinking status, physical activity, TG, HDL cholesterol, and hypertension), except for the stratification variable. Reported p-values were corrected for multiple comparisons. BMI, body mass index; CI, confidence interval; HDL, high-density lipoprotein; HOMA-IR, homeostatic model assessment–insulin resistance; HR, hazard ratio; SAT, subcutaneous adipose tissue; T2D, type 2 diabetes; TG, triglyceride; Waist C., waist circumference. (TIF) [file pmed.1003700.s014.tif]

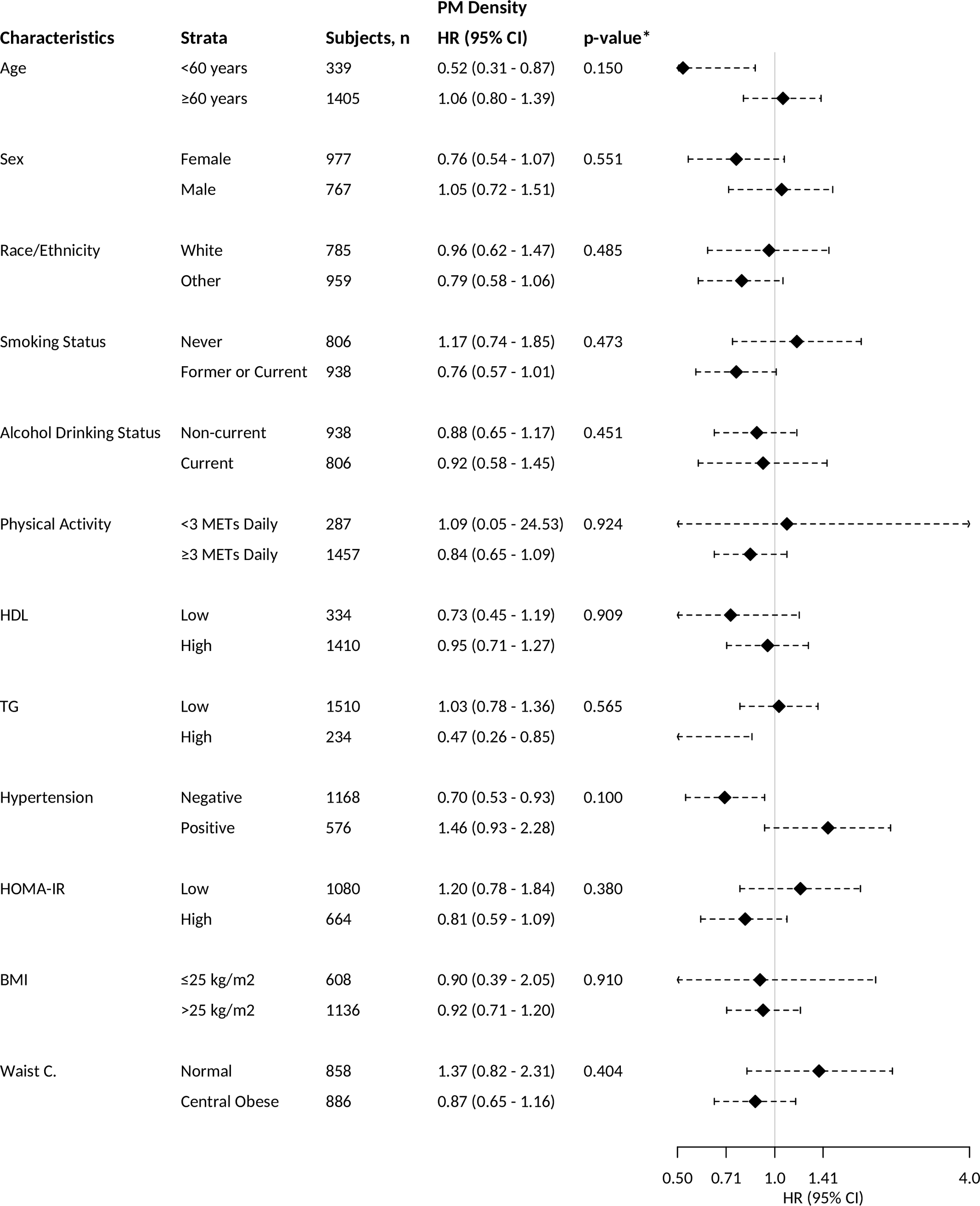

Supplement: S9 Fig — *p-value for interaction. Models were adjusted for covariates in Model 1 (categorical age, sex, race/ethnicity, smoking status, alcohol drinking status, physical activity, TG, HDL cholesterol, and hypertension), except for the stratification variable. Reported p-values were corrected for multiple comparisons. BMI, body mass index; CI, confidence interval; HDL, high-density lipoprotein; HOMA-IR, homeostatic model assessment–insulin resistance; HR, hazard ratio; PM, pectoralis muscle; SD, standard deviation; T2D, type 2 diabetes; TG, triglyceride, Waist C., waist circumference. (TIF) [file pmed.1003700.s015.tif]
