# Supplementary material for: Adipose tissue biomarkers and type 2 diabetes incidence in normoglycemic participants in the MESArthritis Ancillary Study: A cohort study
Source: PLoS Med. 2021 Jul 9;18(7):e1003700. doi: 10.1371/journal.pmed.1003700 (PMC8337053; doi:10.1371/journal.pmed.1003700)
Supplement: Attachment — Submitted filename: Plos_Medicine-D-00345-1.docx [file pmed.1003700.s016.docx]

**Manuscript Number: PMEDICINE-D-00345**

**Title:** Adipose tissue biomarkers and risk of type 2 diabetes incidence in normoglycemic participants: The MESArthritis Ancillary Study

**Review**

A SUMMARY OF THE STUDY

This manuscript entitled “Adipose tissue biomarkers and risk of type 2 diabetes incidence in normoglycemic participants: The MESArthritis Ancillary Study” provides a thorough investigation of the importance of CT-derived markers for adipose tissue and lipids for the incidence of type 2 diabetes in 1,727 normoglycemic study participants, with an average follow-up time of approximately 7 years.

At baseline, CT-derived adipose tissue biomarkers and pectoralis muscle lipids were examined, along with other metabolic factors, anthropometric measures and classical clinical factors, to assess the predictive ability of these covariates for the development of type 2 diabetes or pre-diabetes.

The authors used Cox proportional hazards model to study the research question and 75 individuals developed type 2 diabetes during the follow-up time. The authors also performed ancillary analyses that are of interest for the research question.

Remarks to the Author:

The research question is important and the authors provide interesting data to assess this. A strength of the study is the long follow-up time. Although many study participants are included, only a fraction actually developed events during the study period. I have some general questions regarding study design and some concerns about the statistical modeling discussed below.

|  | **STUDY DESIGN AND RESULTS – COMMENTS** |
| --- | --- |
|  | 1. The authors conclude that intermuscular fat (IMAT), but not subcutaneous fat, has a predictive value for risk of developing T2D also after adjusting for BMI and waist circumference. However, causality is not proven and increased intermuscular fat can just be a marker of other ectopic fat depots including liver fat and NAFLD/NASH which also predict risk of T2D. This should be discussed. 2. The authors mention in the Methods section that IMAT (extramyocellular lipid content), was measured and from this the authors could calculate the intramyocellular lipid content. However, have the authors constructed Cox models for both extra- and intramyocellular lipid content, respectively? I was not able to identify models for both forms of IMAT. |
|  | 3. In the Methods section, the authors state that they measured between 2008 and 2010 baseline values for adipose tissue markers from CT examination, classical clinical characteristics, anthropometric measures and HOMA-IR. The authors also mention that these variables were measured again after 9 to 12 months and that a third measurement was performed at the end of the study period. Does this mean that the authors have serial data or time-updated data for all the mentioned covariates? If so, I would suggest redoing the analyses with time-updated mean values in the Cox models, instead of baseline values. |
|  | 4. On line 157: According to authors, there are 17 study participants with self-reported type 2 diabetes, but without information on insulin treatment or oral- antidiabetic treatment. Is it possible that these individuals were treated with lifestyle interventions (i.e. improved nutritionary intake and increased physical activity)?   There is a considerable number of T2D patients treated with life-style interventions, ranging between 15-35%.  Also, the authors have performed both a supplementary analysis for individuals who developed pre-diabetes and a sensitivity analyses for those that had self-reported T2D. I would not exclude these from the main analyses, if these 17 patients had received a diagnosis of T2D based on fasting plasma glucose levels. |
|  | 5. On line 173: Why did the authors use quantiles for IMAT and SAT? Did they use quantiles for the independent covariates, because they had previously modeled all independent variables as linear covariates and discovered that covariates become insignificant?   The authors have used smoothness function for the Cox model to study the pattern of association for some linear covariates. I would suggest that the authors include restricted cubic splines as a regression modelling strategy, with 4 or 5 evenly spaced knots, for all linear covariates in their regression model. This would result in a superior assessment of the covariates and their nonlinear association with incident T2D. |
|  |  |
|  | 6. On line 216: Do the authors consider the overall incidence of T2D in this study to be generalizable to the general population? It is surprising that only 4.3 % of study participants who were 69 years of age, developed T2D during the follow-up time. I would view this incidence as quite low considering the mean age for this study cohort. The incidence of T2D in this age group is nearly 10% in most western populations. Could there be a reason for a such a low incidence? |
|  |  |
|  | 7. According to Table 2 and as suggested by the authors, the models indicate that there is not a linear association between IMAT- and SAT levels. Therefore, the authors should include a brief paragraph in the discussion regarding this nonlinear association and why the highest quintiles of IMAT and SAT are statistically significant. |
|  |  |
|  |  |
|  | 8. This study has not presented data on comorbidities for study participants. Does the cohort gather information on baseline comorbidities, if so, these should be adjusted for along with other cardiometabolic treatments, such as antihypertensives and statins, since these covariates could have modifying effect on the risk of developing T2D. |
|  | 9. Figure 2 should include a box where color for boxes and dots are descripted rather than in legend text. There is a red dot missing in the first stratified model in Figure 2 in the age strata for >60 |
|  | 10. Why are the models not adjusted for LDL-C? Does the MESA register not include this variable? |
|  | 11. From Figure 2, it seems that IMAT interacts with BMI, increasing age and certain ethnicities while SAT interacts with HOMA-IR, sex and ethnicity. It is an interesting aspect that IMAT interacts with older age but high SAT in young individuals was associated with increasing T2D incidence. The authors should discuss this and any possible reasons.. |
|  | 12. In Supplementary Table S6: How do you explain that IMAT and SAT were not predictable of pre-diabetes but of incident T2?. Is it simply a case of insufficient events in the statistical models? |
|  | 13. Supplementary Figure S5: This figure is difficult to interpret. How was missing data handled in the cox models? Has this something to do with the nested cox-model, which is mentioned in the Methods section? A nested approach to Cox models could mean different things and I assume that it has to do with the nested approach to missing data in the cox proportional hazards model? The authors should have imputed missing data, with either MICE or a machine learning approach, which is considered standard approach for imputing missing values today. If the prevalence of missing values is low then imputation with mean values could be acceptable. What is the y-axis in Figure S5, proportion or numbers? |

Confidential Conflict of Interest (Required):
None to declare.
