# Supplementary material for: Adipose tissue biomarkers and type 2 diabetes incidence in normoglycemic participants in the MESArthritis Ancillary Study: A cohort study
Source: PLoS Med. 2021 Jul 9;18(7):e1003700. doi: 10.1371/journal.pmed.1003700 (PMC8337053; doi:10.1371/journal.pmed.1003700)
Supplement: Attachment — Submitted filename: renamed_bf73f.docx [file pmed.1003700.s017.docx]

Dear editor and reviewers

Thank you so much for reviewing our manuscript. The authors are grateful for the comments. We carefully read the comments and tried to comply with all requests as you can see in the revised version. In this regard, we undertook major changes to comprehensively address the comments. The major changes include:

a. The main text is reviewed and the language that implied causality is removed,
b. The missing data points are multiply imputed to produce 5 datasets, each dataset is analyzed separately, and the results of analyses were pooled across the datasets,
c. The associations between adipose tissue biomarkers and diabetes incidence are now reported per 1-SD increment in the biomarker, and
d. The reported p-values are corrected for multiple comparisons.

A point-by-point rebuttal letter to editor and reviewers and reference to revisions for every comment are included below.

To improve the navigation through the letter, we have used color code for the editor as blue (E), reviewer-1 as green (R1), reviewer-2 as red (R2), and reviewer-3 as orange (R3) both in the letter and the annotated version of the main text.

In the end, I would like to thank you for your precious time and scientific comments.  Please do not hesitate to inform us in case of any additional comments.

Sincerely,

Shadpour Demehri MD,

The Russell H. Morgan Department of Radiology and Radiological Science,

Johns Hopkins University School of Medicine,

601 N Caroline St, JHOC 3171c, Baltimore, MD 21287;

Telephone: (410)-416-4179; Fax: (410)-502-6454; Email: Demehri2001@Yahoo.com

***Editors***

1. Please revise your title according to PLOS Medicine's style. Your title must be nondeclarative and not a question. It should begin with main concept if possible. "Effect of" or “risk of” should be used only if causality can be inferred, i.e., for an RCT. Please place the study design ("A retrospective study,") in the subtitle (i.e., after a colon). For example, “Adipose tissue biomarkers and type 2 diabetes incidence in normoglycemic participants in The MESArthritis Ancillary Study: A retrospective cohort study”

**Answer**

*The title of the study has been updated as requested.*

**Action**

*Title – Line 2*

*The title of the study has been updated to:*

*“Adipose tissue biomarkers and type 2 diabetes incidence in normoglycemic participants in The MESArthritis Ancillary Study: A retrospective cohort study”.*

2. In the Data Availability Statement, please provide a direct email address or more direct web link which can be used to access the data. Clicking on the provided link, it is not clear which steps need to be taken to request data access.

**Answer**

*The requested information has been added to the Data Availability Statement.*

**Action**

*Data Availability Statement*

*The requested information has been added.*

3. (Abstract) Please ensure that all numbers presented in the abstract are present and identical to numbers presented in the main manuscript text.

**Answer**

*The numbers presented in the abstract have been rechecked to be identical to the numbers presented in the main text.*

**Action**

*Abstract – Throughout*

*The numbers presented in the abstract have been rechecked to be identical to the numbers presented in the main text.*

4. (Abstract) Please include the study design, population and setting, number of participants, years during which the study took place, length of follow up, and main outcome measures.

**Answer**

*The requested information has been added to the abstract.*

**Action**

*Abstract – Methods and Findings – Line 46*

*The requested information has been added to the abstract. This part reads as:*

*“This study was a population-based multi-ethnic retrospective cohort of 1,744 participants in the Multi-Ethnic Study of Atherosclerosis (MESA) with normoglycemia (baseline fasting plasma glucose less than 100 mg/dL) from 6 United States communities. Participants were followed from April 2010 and January 2012 to December 2017, for a median of 7 years. The intermuscular (IMAT) and subcutaneous (SAT) adipose tissue areas were measured in baseline chest CT exams and were corrected by height squared (SAT and IMAT indices) using a predefined measurement protocol. T2D incidence, as the main outcome, was based on follow-up fasting plasma glucose, review of hospital records, or self-reported physician diagnoses.”*

5. (Abstract) Please include the actual amounts and/or absolute risk(s) of relevant outcomes, not just relative risks or correlation coefficients

**Answer**

*The requested information has been added to the abstract.*

**Action**

*Abstract – Methods and Findings – Line 54*

*The requested information has been added to the abstract. This part reads as:*

*“Participants' mean age was 69 ± 9 years at baseline, and 977 (56.0%) were women. Over a median of 7 years, 103 (5.9%) participants were diagnosed with T2D and 147 (8.4%) participants died.”*

6. (Abstract) Please include a summary of adverse events if these were assessed in the study.

**Answer**

*The requested information has been added to the abstract.*

**Action**

*Abstract – Methods and Findings – Line 54*

*The requested information has been added to the abstract. This part reads as:*

*“Participants' mean age was 69 ± 9 years at baseline, and 977 (56.0%) were women. Over a median of 7 years, 103 (5.9%) participants were diagnosed with T2D and 147 (8.4%) participants died.”*

7. (Abstract) In the last sentence of the Abstract Methods and Findings section, please describe the main limitation(s) of the study's methodology.

**Answer**

*The requested information has been added to the abstract.*

**Action**

*Abstract – Methods and Findings – Line 61*

*The requested information has been added to the abstract. This part reads as:*

*“The limitations of this study include unmeasured residual confounders and one-time measurement of adipose tissue biomarkers.”*

8. (Abstract) Abstract Conclusions: Please address the study implications without overreaching what can be concluded from the data; the phrase "In this study, we observed ..." may be useful. Please interpret the study based on the results presented in the abstract, emphasizing what is new.

**Answer**

*The language of the conclusions of the abstract has been attenuated and updated as requested.*

**Action**

*Abstract – Conclusions – Line 64*

*The language of the abstract has been attenuated and updated as requested. This part reads as:*

*“In this study, we observed an association between intermuscular adipose tissue at baseline and T2D incidence over the follow-up. This study suggests the potential role of intermuscular adipose depots in the pathophysiology of T2D.”*

9. Please conclude the Introduction with a clear description of the study question or hypothesis.

**Answer**

*The requested information has been added to the introduction.*

**Action**

*Introduction – Line 119*

*The requested information has been added to the introduction. This part reads as:*

*“In the present study, we used the population-based multi-ethnic cohort of participants in the Multi-Ethnic Study of Atherosclerosis (MESA) to assess the associations between CT-derived adipose depots biomarkers and T2D incidence. We characterized intermuscular adipose tissue (IMAT), subcutaneous adipose tissue (SAT), and intramyocellular lipid contents in the baseline chest CT exams, and studied the potential associations between these biomarkers and T2D incidence.”*

10. For this observational study, in the manuscript text, please indicate: (1) the analytical methods by which you planned to test your hypothesis, (2) the analyses you actually performed, and (3) when reported analyses differ from those that were planned, transparent explanations for differences that affect the reliability of the study's results. If a reported analysis was performed based on an interesting but unanticipated pattern in the data, please be clear that the analysis was data-driven.

**Answer**

*The requested information has been added to the methods. Moreover, the S2 Table has been added to summarize the planned and operationalized approaches in the statistical analyses of this work.*

**Action**

*Methods – Line 183*

*In addition to adding the S2 Table, the following paragraph has been added to methods:*

*“The statistical analyses of this study were planned a priori at the time of preparing the research proposal and was approved by the MESA publication and steering committees. Few additions (e.g., assessing the linearity of the associations between adipose tissue biomarkers and T2D incidence and multiply imputing the missing data points) or changes (e.g., reporting the associations per 1-standard deviation [1-SD] increment in the biomarkers) were made to the planned statistical analyses at the time of data analysis or per peer-reviewers comments (S2 Table).”*

11. Did your study have a prospective protocol or analysis plan? Please state this (either way) early in the Methods section.

a) If a prospective analysis plan (from your funding proposal, IRB or other ethics committee submission, study protocol, or other planning document written before analyzing the data) was used in designing the study, please include the relevant prospectively written document with your revised manuscript as a Supporting Information file to be published alongside your study, and cite it in the Methods section. A legend for this file should be included at the end of your manuscript.

b) If no such document exists, please make sure that the Methods section transparently describes when analyses were planned, and when/why any data-driven changes to analyses took place.

c) In either case, changes in the analysis-- including those made in response to peer review comments-- should be identified as such in the Methods section of the paper, with rationale.

**Answer**

*The manuscript proposal of this paper, which included the details of study design and statistical analyses, was approved a priori by the MESA publication and steering committees. Although (per MESA policies) we cannot share the manuscript proposal, we have summarized the planned and operationalized approaches of statistical analyses in the S2 Table.*

**Action**

*Methods – Line 183*

*The S2 Table has been added to the supporting information to highlight the few additions (e.g., assessing the linearity of the associations between adipose tissue biomarkers and diabetes incidence and multiply imputing the missing data points) or changes (e.g., reporting the associations per 1-standard deviation [1-SD] increment in the biomarkers) that were made to the planned statistical analyses at the time of data analysis or per peer-reviewers comments.*

12. Please ensure that the study is reported according to the STROBE guideline, and include the completed STROBE checklist as Supporting Information. Please add the following statement, or similar, to the Methods: "This study is reported as per the Strengthening the Reporting of Observational Studies in Epidemiology (STROBE) guideline (S1 Checklist)." The STROBE guideline can be found here: http://www.equator-network.org/reporting-guidelines/strobe/

**Answer**

*The STROBE checklist is now added to S1 Checklist.*

**Action**

*Methods – Statistical Analysis – Line 182*

*The STROBE checklist has been added, with a citation in the main text as:*

*“This study is reported as per the Strengthening the Reporting of Observational Studies in Epidemiology (STROBE) guideline (S1 Checklist).”*

13. Your study is observational and therefore causality cannot be inferred. Please remove language that implies causality, such as - predict, risk, effect, etc. Instead, refer to associations consistently throughout the text.

**Answer**

*The main text has been reviewed several times and the languages that imply causality has been removed.*

**Action**

*Main Text – Throughout*

*The main text has been reviewed several times and the languages that imply causality has been removed.*

14. Please define the length of follow up (e.g., in mean, SD, and range).

**Answer**

*The requested information has been added to the methods and results.*

**Action**

*Methods – T2D Diagnosis – Line 150*

*Results – Baseline Characteristics – Line 238*

*The requested information has been added to the methods and results. In the methods – T2D diagnosis:*

*“Between baseline (April 2010 to January 2012) to December 2017 …”*

*In the* *results – baseline characteristics:*

*“Participants were followed for a median of 6.8 [6.2 - 7.2] years, and during this period, 103 (5.9%) participants were diagnosed with T2D and 147 (8.4%) participants died (137 [7.9%] without T2D, Fig 1).”*

15. How was race/ethnicity defined and by whom? Why race/ethnicity was considered important in this study and what it is believed to represent e.g., are SES or genetic differences being attributed to race/ethnicity?

**Answer**

*Thanks for the comment.*

*The MESA or Multi-Ethnic Study of Atherosclerosis is a prospective study of the characteristics of cardiovascular disease and the risk factors that predict the progression of diseases to clinically overt cardiovascular disease. MESA includes a diverse, population-based sample of men and women aged 45-84 from six field centers across the United States, who identified themselves as white, black, Hispanic, or Chinese-American. Approximately 38% of the recruited participants were white, 28% black, 22% Hispanic, and 12% Chinese-American. The MESArthritis includes approximately half of the participants in the MESA (who completed the 5^th^ MESA exam from April 2010 to January 2012, 3,083 out of 6,814) with similar race/ethnicity distribution (39% white, 28% black, 21% Hispanic, and 12% Chinese-American).*

*Although it is beyond the scope of our study, the MESA was aimed to recruit participants similar to the United States population racial/ethnic distribution. The study recruited fewer Chinese-American participants than what was anticipated, however, the distribution of participants with white, black, and Hispanic race/ethnicity was comparable to the United States population structure at the time. It is believed that race/ethnicity is linked both to genetic differences and socioeconomic factors. Two research groups inside the network of MESA collaborators; “genetic ancestry” and “race/ethnicity and events,” are currently working on these topics.*

**Action**

*N/A*

16. Please indicate and discuss whether your findings were validated in a different data set.

**Answer**

*Thanks for the comment.*

*We have not validated our findings in a different dataset, as there is no other prospective cohort study that includes both chest CT exam at baseline and a thorough follow-up of participants for diabetes incidence available to us. However, other studies used other imaging techniques such as abdomen CT exam to assess the associations between adipose tissue biomarkers and diabetes incidence (over a shorter follow-up and usually without time-to-event analysis).*

*The novelty of our work was the use of the chest CT exam for measuring the adipose tissue biomarkers. The chest CT exam is performed routinely in the clinical practice as a screening tool for common cardiopulmonary disorders such as lung cancer screening and calcium scoring and availability of these exams provide the opportunity to extract information on adipose tissue biomarker at zero additional radiations or costs.*

**Action**

*Discussion – Throughout*

*We reviewed the discussion and add relevant language to compare the findings of our study to previously published studies in this field.*

17. In statistical methods, please refer to any post-hoc corrections to correct for multiple comparisons during your statistical analyses. If these were not performed, please justify the reasons. Please refer to our statistical reporting guidelines for assistance (https://journals.plos.org/plosone/s/submission-guidelines.#loc-statistical-reporting)

**Answer**

*The reported p-values were corrected using the Benjamini-Hochberg procedure. Details of this process are now added to methods.*

**Action**

*Methods – Statistical Analysis – Line 229*

*The reported p-values were corrected using the Benjamini-Hochberg procedure. Details of this process are now added to methods and read as:*

*“We applied the Benjamini–Hochberg procedure to correct the p-values for multiple comparisons. The p-values from main, sensitivity, and supplementary analyses were batched and corrected separately using this procedure.[28] The associations with p-values <0.05 were considered statistically significant.”*

18. We note that your data are clustered at hospital and regional levels. Please elaborate how your account for clustering in your statistical methods.

**Answer**

*Thanks for the comment.*

*MESA includes a population-based sample of men and women from six field centers across the United States. In addition to the field center of the study, the race/ethnicity bears (to an acceptable extent) information on the site of the study. Authors acknowledge the (possible) clustered structure of the dataset at the field center levels, however, following the “MESA Analysis Recommendations”, we should include the “field center” or the “race/ethnicity” (not both) in our statistical models, and we selected the “race/ethnicity” for adjusting our models.*

*The table below summarizes the overlap between the “field center” and the “race/ethnicity” in the MESArthritis.*

| *Race/Ethnicity* | *Wake Forest U.* | *Columbia U.* | *Johns Hopkins U.* | *U. of Minnesota* | *Northwestern U.* | *U. of California L.A* |
| --- | --- | --- | --- | --- | --- | --- |
| *White* | *297* | *151* | *220* | *222* | *261* | *52* |
| *Black* | *256* | *196* | *203* | *0* | *128* | *62* |
| *Hispanic* | *0* | *302* | *0* | *183* | *0* | *169* |
| *Chinese-American* | *0* | *2* | *0* | *0* | *150* | *229* |

**Action**

*N/A*

19. Please describe how you selected your adjustment variables.

**Answer**

*Thanks for the comment.*

*The covariates used for adjusting the statistical models were selected based on the available literature on this topic. For instance, the paper by Flowers et al. used exactly the same covariates for adjusting the statistical models as our study (the paper was about the association between body composition and diabetes incidence in South Asians).*

*We have added a citation to the reference that we used for selecting the covariates in the statistical models.*

**Action**

*Methods – Statistical Analysis – Line 204*

*We have added a citation to the reference that we used for selecting the covariates in the statistical models.*

20. We note the potential for unobservable confounding in this observational study. Please consider using robust methods such as propensity score matching to address this.

**Answer**

*Thanks for the comment. The authors acknowledge the potential effects of residual confounding and have emphasized this issue in the first limitation of the study in the discussion. However, we should mention that given the selected covariates in the models [based on existing literature on this topic] and the quality of the collected data in the MESA [i.e., relatively low missing data points and consistent data collection protocols], the residual confounder effect on the reported associations is probably low.*

*Authors acknowledge the use of robust methods such as propensity score matching to address limitations of observational studies. Although there are some new methods to use propensity score matching when the exposure (treatment) variable is continuous (like in our study, the adipose tissue biomarkers), methods like propensity score matching are most useful when the exposure variables are dichotomous.*

*Given the nature of the exposure variables (continuous or categorized using quartiles) in our study, we performed propensity score-weighting to balance effects of all covariates (traditional risk factors of diabetes, HOMA-IR, BMI, and waist circumference) across the 4 groups of (quartiles of) exposure variables (IMAT index, SAT index, and PM density, in this analysis missing data points were not imputed and extreme weights are trimmed at 95%).*

|  | ***Quartile 1*** | ***Quartile 2**** | ***Quartile 3**** | ***Quartile 4**** |
| --- | --- | --- | --- | --- |
| ***IMAT Index***  *HR (95% CI), p-value* | *1 (reference)* | *1.66 (1.12 - 2.46), 0.012* | *1.68 (2.48 - 1.13), 0.010* | *2.43 (3.58 - 1.65), <0.001* |
| ***SAT Index***  *HR (95% CI), p-value* | *1 (reference)* | *1.03 (0.67 - 1.57), 0.891* | *1.27 (1.90 - 0.85), 0.238* | *1.38 (2.12 - 0.90), 0.135* |
| ***PM Density***  *HR (95% CI), p-value* | *1 (reference)* | *0.97 (0.71 - 1.32), 0.837* | *0.93 (1.27 - 0.68), 0.666* | *1.03 (1.44 - 0.73), 0.871* |

** p-values are not corrected*

*We would like to mention that methods like propensity score matching and weighting may lower effects of residual confounders, but cannot fully balance the study groups for variables not observed or not included in the models.*

**Action**

*N/A*

21. Please write “95% CI” when you provide estimates and 95% CI

**Answer**

*The text has been reviewed and the requested details have been added.*

**Action**

*Main Text – Throughout*

*The text has been reviewed and the requested details have been added.*

22. Please specify the significance level used (e.g., P<0.05, two-sided) and the statistical test used to derive a p value.

**Answer**

*The requested information has been added to the methods.*

**Action**

*Methods – Statistical Analysis – Line 231*

*The requested information has been added to the methods as:*

*“The associations with p-values <0.05 were considered statistically significant.”*

23. Please provide the full name(s) of the institutional review board(s) that provided ethical approval.

**Answer**

*The requested information has been added to the methods. More details are listed below:*

*Wake Forest University	IRB number IRB00008492 under Federal-wide Assurance - FWA00001435*

*Columbia University	IRB number IRB00002973 under Federal-wide Assurance - FWA00002636*

*Johns Hopkins University	IRB number 00001656 under Federal-wide Assurance - FWA00005752*

*University of Minnesota	IRB number IRB00000438 under Federal-wide Assurance - FWA00000312*

*Northwestern University	IRB number IRB00005003 under Federal-wide Assurance - FWA00001549*

*U. of California L.A	IRB number 00000172 under Federal-wide Assurance - FWA00004642*

*U. of Washington	IRB number IRB00005647 under Federal-wide Assurance - FWA00006878*

**Action**

*Methods – Line 129*

*The required information has been added to the methods as:*

*“The MESA was approved by the institutional review boards of the six participating field centers (Columbia University, Johns Hopkins University, Northwestern University, University of California, University of Minnesota, and Wake Forest University) and the coordinating center (University of Washington).”*

24. For S3 figure of the chest CT, please ensure that you have complied with our figures requirements http://journals.plos.org/plosmedicine/s/figures.

**Answer**

*The S3 Fig has been updated to comply with PLOS Medicine figures requirements.*

**Action**

*S3 Fig*

*The S3 Fig has been updated to comply with PLOS Medicine figures requirements.*

25. Please replace “nondiabetics” with “persons without diabetes.”

**Answer**

*The text has been reviewed and updated as requested.*

**Action**

*Main Text – Throughout*

*The text has been reviewed and updated as requested.*

26. Please replace "Caucasian" with "white" throughout the paper.

**Answer**

*The text has been reviewed and updated as requested.*

**Action**

*Main Text – Throughout*

*The text has been reviewed and updated as requested.*

***Reviewer #1***

The authors examined the associations of CT-measured indices with incident type 2 diabetes (T2D). This study has a novel component of the use of CT-derived variables. Its contributions to science and clinical practice may be meaningful, while the reviewer is not an expert on it. While positive remarks are possible, some concerns are present as described in the following paragraphs, Major Comments and Minor Comments.

**Major Comments**

0. The authors identified a significant interaction by BMI for the primary finding. Thus, the average association presented in the abstract and the main results is not meaningful. The abstract and "Author Summary" should clarify that the positive association was present among those with overweight or obesity (BMI>24.9).

The authors need to assess the association stratified by BMI with statistical adjustment for BMI (within stratum) and waist circumference. It is not clear if the authors did it.

**Answer**

*Thanks for the comment.*

*Per reviewers’ comments, we have made several changes to our approach in statistical analysis, including 1. Multiply imputing the missing data points before Cox proportional hazard modeling, 2. Reporting the association between adipose tissue biomarkers and diabetes incidence per 1-SD increment in the biomarkers (instead of log2-transformed analysis), 3. Correcting the reported p-values for multiple comparisons, and 4. Changing the strata in the BMI-stratified analyses from BMI ≤24.9 vs >24.9 to BMI ≤25.0 vs >25 kg/m2.*

*Following these changes, the reported p-value for the interaction between BMI and IMAT index (in the stratification analysis) is no longer “significant”. Although we have updated and attenuated the language in the abstract, author summary, and discussion to reflect the reviewer’s concerns, we tried not to discuss the “insignificant” p-value for interaction between BMI and IMAT index.*

*Regarding the reviewer’s comment on including the BMI and waist circumference in the BMI-stratified analyses, results “with and without” BMI and waist circumference in the models are presented below:*

|  | ***Strata*** | ***Without BMI and Waist Circumference in the Models***  *HR (95% CI)* | ***With BMI and Waist Circumference in the Models***  *HR (95% CI)* |
| --- | --- | --- | --- |
| *IMAT Index* | *≤25 kg/m2* | *0.97 (0.39 – 2.37)* | *1.02 (0.43 – 2.42)* |
|  | *>25 kg/m2* | *1.26 (1.13 – 1.39)* | *1.23 (1.09 – 1.38)* |
| *SAT Index* | *≤25 kg/m2* | *1.45 (0.54 – 3.89)* | *1.62 (0.55 – 4.76)* |
|  | *>25 kg/m2* | *1.35 (1.06 – 1.71)* | *1.22 (0.89 – 1.67)* |
| *PM Density* | *≤25 kg/m2* | *0.90 (0.45 – 1.79)* | *0.89 (0.43 – 1.86)* |
|  | *>25 kg/m2* | *0.92 (0.71 – 1.19)* | *0.97 (0.73 – 1.28)* |

*The authors acknowledge the reviewer’s concern, however, given the little effects of including BMI and waist circumference in the models, the authors did not include these results in the main text.*

**Action**

*N/A*

1. The authors should revise the description of the results throughout the manuscript. The authors dichotomized their inference into two mainly: significant or not. That is malpractice in recent medical science. The authors should avoid it throughout the manuscript.

Accounting for no perfect measure of central or whole-body adiposity, the authors need to keep the following interpretations:

i) There were associations of the SAT and IMAT indices with T2D among the participants with overweight or obesity.

ii) The association was partly related to insulin resistance and anthropometric measures.

iii) The intermuscular fat related to the development of T2D. Thus, this study demonstrated that the possible failure of storing fat intracellularly in adults with overweight or obesity could be the consequence, pathological process, or both of the development of T2D.

The authors should avoid describing that one type of association was independent of the adiposity or that the IMAT index predicted T2D. This kind of interpretation would be too strong given the observational analyses with measurement errors. Of note, even if the association became null after adjustment for BMI and waist circumference, this study seems to be pathophysiologically important. However, for the particular argument, interaction by overweight or obesity status is essential to highlight.

**Answer**

*The languages in the abstract, author summary, results, and discussion are updated to reflect the reviewer’s concerns. Although we did not discuss the “insignificant” p-values for the interaction between BMI and IMAT index, we tried to address other issues raised in this comment.*

**Action**

*Abstract – Conclusion – Line 64*

*Authors Summary – What do these findings mean? – Line 83*

*Results – Throughout*

*Discussion – Throughout*

*The text has been reviewed several times to avoid over-emphasizing p-values and address the reviewer’s comments.*

2. The introduction and discussion are insufficient.

The authors should cite several genetic, Mendelian randomization studies relating anthropometric measures to T2D, such as the one in JAMA (LA Lotta et al., JAMA, 2018). Genetic evidence is already available to indicate that fat storage and its location matter in the development of T2D. The authors should present it as background information for this work and discuss the pathophysiology of T2D.

**Answer**

*We re-wrote the introduction and tried to address the concerns raised in this comment. We added citations to 3 recent (GWAS and imaging assessments) studies which suggested associations between ectopic adipose depots and diabetes incidence. However, these studies (including the one mentioned in this comment) showed the link between ectopic adipose depots in the liver and visceral organs, while in our paper, we are interested in adipose depots in muscles (owing to the skeletal muscles cardinal role in glucose homeostasis). We added these studies to our introduction.*

**Action**

*Introduction – Line 97*

*We re-wrote the introduction and tried to address the concerns raised in this comment. This part now reads as:*

*“There are known associations between obesity or excessive overall adipose depots and the T2D incidence.[3-5] However, recent data from genome-wide association studies [6, 7] and imaging assessments [7] suggest a stronger association between ectopic adipose depots (in liver, visceral organs, and muscles) and T2D incidence (compared to excessive overall adipose depots).[8-10] Specifically, adipose depots deposition beneath the fascia of skeletal muscles (extramyocellular and intramyocellular lipid content) may contribute to T2D incidence, owing to the skeletal muscles cardinal role in glucose homeostasis.[11]The body mass index (BMI) and other clinical anthropometric indices, despite quantifying the excessive overall adipose depots, do not provide data on ectopic adipose depots distribution.”*

3. The reviewer has considered the pathophysiological importance of this work. The authors emphasized it in the introduction and because the reviewer has recognized the sparsity of the relevant evidence in large-scale epidemiological research. However, the authors may eagerly want to establish the importance of the CT-derived measures to predict T2D risk regardless of the etiological pathway. If so, the authors should revise the introduction to explain why the prediction modelling for T2D should be improved, citing previous studies on T2D prediction (e.g., ones from Framingham Offspring Study, ARIC, MESA). The authors have not done it, leaving the argument about prediction unconvincing or not structured well.

Moreover, if the authors are interested in prediction, its evaluation requires refinement. The authors mainly interpreted whether the measures of associations were significant or not. This interpretation was substandard for its utility for the prediction. A further quantitative evaluation scoping possible clinical actions will be essential (see Cook et al., Annals Intern Med, 2009; 150(11):795-802).

Finally, if the authors want to argue the utility of a CT exam for the prediction of T2D, the evaluation should include other CT results in addition to the chest ones. The emphasis for prediction would not make sense in this manuscript.

**Answer**

*The authors acknowledge the concerns of the reviewer. We reviewed the discussion, attenuated the tone in this part, and tried to remove the language that implied “establishing the importance of the CT-derived measures to predict T2D risk”. Specifically, we removed all the keywords that implied causality (e.g., risk and predict) and also focused on the pathological pathway behind the association between IMAT and diabetes incidence.*

*However, we kept the few sentences that showed the CT exams we used for our study were primarily performed for other initial indications and also how our finding may extend the value of performing CT exams recommended in clinical guidelines.*

**Action**

*Discussion – Throughout*

*The text has been reviewed and updated as requested.*

4. S6 Fig is incorrect. Hazard ratios cannot be negative. If an author estimates incident rate ratio or hazard ratio, one reference point without any standard errors should be available. The current figure does not have it. A valid explanation is necessary if the figure is valid.

**Answer**

*The y-axis caption in this figure was incorrect. The caption has been updated and the mistake is rectified as requested.*

**Action**

*S6 Fig*

*The y-axis caption has been updated and the mistake is corrected. The new captions are:*

*s(IMAT Index, 1.05) in panel A, s(SAT Index 1.00) in panel B, and s(PM Density , 1.00) in panel C.*

**Minor Comments**

5. The primary results are owing to an assessment of statistical significance applied to p-value=0.036. Given the number of tests operated in this study (for the two CT measures, for instance), the inference based on the p-value is inappropriate. As generally recommended against the dichotomic use of p-value, the authors should revise the manuscript to judge the results based on statistical significance.

**Answer**

*Thanks for the comment.*

*As described in the answer to comment 0, we have made several changes to our approach in statistical analysis, including reporting the association per 1-SD increment in the biomarkers, multiply imputing the missing data points, and correcting the reported p-values for multiple comparisons (among others). The mentioned p-value in this comment (as a result of changes to the statistical analyses), is now p-value <0.001 – after correction for multiple comparisons.*

*The authors acknowledge the reviewer’s concern in dichotomizing the p-values and have reviewed the main text and updated the language in the main text accordingly. However, per the editor’s comments, we kept and reported the p-values in the main text.*

**Action**

*Main text – Throughout*

*The text has been reviewed several times to avoid over-emphasizing p-values and address the reviewer’s comments.*

Abstract:

6. Background: "and contribute to" should be deleted. After the deletion, the sentence still works, and it will turn out to be sharp and straightforward.

**Answer**

*The text has been reviewed and updated as requested.*

**Action**

*Abstract – Background – Line 40*

*The text has been reviewed and updated as requested. This part now reads as:*

*“Given the central role of skeletal muscles in glucose homeostasis, deposition of adipose depots beneath the fascia of muscles (versus subcutaneous adipose tissue) may precede insulin resistance and type 2 diabetes (T2D) incidence.”*

7. Calendar years of the follow-up start and finish should be available in the abstract. The MESA started around 2000, but this particular study's baseline started around 2010 from the CT measurements. Thus, for some readers, years should be documented.

**Answer**

*The text has been reviewed and updated as requested.*

**Action**

*Abstract – Methods and Findings – Line 48*

*The text has been reviewed and updated as requested. This part now reads as:*

*“Participants were followed from April 2010 and January 2012 to December 2017, for a median of 7 years. The intermuscular (IMAT) and subcutaneous (SAT) adipose tissue areas were measured in baseline chest CT exams and were corrected by height squared (SAT and IMAT indices) using a predefined measurement protocol.”*

8. Use "T2D" after spell out once the authors explain the abbreviation throughout the abstract.

**Answer**

*The text has been reviewed and updated as requested.*

**Action**

*Abstract – Throughout*

*The text has been reviewed and updated as requested.*

9. The authors should not provide a positive or negative connotation based on statistical significance. The authors have stated, "The association of the IMAT index remained statistically significant after adjusting for body mass index and waist circumference (HR: 1.30 [95%CI: 1.02-1.65]), however, the SAT index was no longer associated with type 2 diabetes incidence." This sentence should be revised as follows: "The associations of the SAT and IMAT indices with T2D incidence were attenuated toward the null after adjustment for body mass index and waist circumference, with HR (95% CI) of X.XX (X.XX-X.XX) and 1.30 (1.02-1.65), respectively".

**Answer**

*The text has been reviewed and updated as requested.*

**Action**

*Abstract – Methods and Findings – Line 56*

*The text has been reviewed and updated as requested. This part now reads as:*

*“The IMAT index (HR: 1.27 [95% CI: 1.15-1.41] per 1-standard deviation [SD] increment) and the SAT index (HR: 1.43 [95% CI: 1.16-1.77] per 1-SD increment) at baseline were associated with T2D incidence over the follow-up. The associations of the IMAT and SAT indices with T2D incidence were attenuated after adjustment for body mass index and waist circumference, with HRs of 1.23 (95% CI: 1.09-1.38) and 1.29 (95% CI: 0.96-1.74) per 1-SD increment, respectively.”*

10. Given the possible measurement errors of body mass and waist circumference, the authors should consider a more substantial attenuation after the adjustment and describe the results and interpretation, accounting for the consideration.

**Answer**

*Thanks for the comment.*

*The authors acknowledge the concerns of the reviewer about the measurement errors of body mass index and waist circumference. However, we would like to highlight the fact that for each exam of the MESA, a detailed measurement protocol is prepared and several educational courses for study coordinators in each study site are planned to minimize the measurement errors.*

*After adjusting the statistical models for the BMI and waist circumference, the estimated HRs were attenuated toward the null hypothesis and the estimated confidence intervals were widened (though the estimated HR for the IMAT index remained “statistically significant”).*

*We tried to attenuate the tone of language in the results while discussing results of the models adjusted for BMI and waist circumference.*

**Action**

*Results – Line 249 and 276*

*We tried to attenuate the tone of language in the results while discussing results of the models adjusted for BMI and waist circumference.*

11. The conclusion is too strong and odd. The authors must not be interested in the improvement of prediction via the index they used. Instead, the authors must be interested in how important the intermuscular fat may be in the pathophysiology of T2D. In a sense, the authors may not want to digress the logical flow to a prediction story. The authors may want to state the association partly related to adiposity and highlight the etiological importance of the intermuscular fat. The conclusion requires such a revision.

**Answer**

*The text has been reviewed and updated as requested.*

**Action**

*Abstract – Conclusions – Line 64*

*The text has been reviewed and updated as requested. This part now reads as:*

*“In this study, we observed an association between intermuscular adipose tissue at baseline and T2D incidence over the follow-up. This study suggests the potential role of intermuscular adipose depots in the pathophysiology of T2D.”*

Author Summary:

12. The first text read as if the participants had used time-to-event data. The authors can delete "using longitudinal time-to-event analysis". By saying "baseline" and "incidence", the longitudinal aspect is readable.

**Answer**

*The text has been reviewed and updated as requested.*

**Action**

*Author Summary – Why was this study done? – Line 69*

*The text has been reviewed and updated as requested. This part now reads as:*

*“This study was designed to investigate the associations between computed tomography (CT)-derived biomarkers for adipose tissue at baseline and type 2 diabetes incidence in normoglycemic adults.”*

13. Delete "in this study" and start the bullet point with "We".

**Answer**

*The text has been reviewed and updated as requested.*

**Action**

*Author Summary – What did the researchers do and find? – Line 73*

*The text has been reviewed and updated as requested. This part now reads as:*

*“We assessed 1,744 normoglycemic participants with chest CT exams between 2010 and 2012.”*

14. Line 75: The authors should avoid the dichotomic expression of the results.

**Answer**

*The text has been reviewed and updated as requested.*

**Action**

*Author Summary – Throughout*

*The text has been reviewed several times to avoid over-emphasizing p-values and address the reviewer’s comments.*

Methods

15. Line 186-187: For additive modeling and smoothness estimation, the statistical function should be clear enough for readers to replicate the analysis. There can be different ways.

**Answer**

*The legend of S6 Fig has been updated to include more information about additive modeling.*

**Action**

*S6 Fig*

*The legend of S6 Fig is updated as:*

*“Smooth function estimates (red lines) obtained from ﬁtting a generalized additive Cox proportional hazard models with integrated smoothness estimation on the dataset, with estimated 95% confidence interval (blue lines) is shown. The results are reported on the scale of the adipose tissue biomarkers (per 1-SD increment), and the models were adjusted for covariates in Model 1 (categorical age, sex, race/ethnicity, smoking status, alcohol drinking status, physical activity, TG, HDL cholesterol, and hypertension). The numbers in brackets in the y-axis captions are the estimated degrees of freedom of the smooth curves. The rug marks along the x-axis indicate the adipose tissue biomarkers values.”*

16. Line 188: "consecutive nested" is not necessary unless this phrase means something extraordinary.

**Answer**

*The text has been reviewed and updated as requested.*

**Action**

*Methods – Statistical Analysis – Line 199*

*The text has been reviewed and updated as requested. This part now reads as:*

*“Moreover, several adjusted Cox proportional hazard models were used to estimate the hazard ratios (HRs) and 95% confidence interval (CI) for the T2D incidence according to adipose tissue biomarkers (per 1-SD increment).”*

17. BMI and alcohol can have non-linear associations with T2D incidence. The current approach would be suboptimal. The authors are, in particular, interested in the role of BMI in the evaluation of the association. Therefore, additive modelling for BMI is reasonable in this study.

**Answer**

*Thanks for the comment.*

*The authors acknowledge the potential non-linear associations between BMI and diabetes incidence, as shown in the figure below (models are adjusted for traditional risk factors of diabetes, BMI, and waist circumference, in the models in panel A, B, C, IMAT index, SAT index, and PM density were also included).*

*However, including the smoothed function of the BMI in the models will result in similar HR values as the ones already reported in the main text. In the first column of the table below, the HRs for the association between adipose tissue biomarkers and diabetes incidence (as already reported in the main text) are compared to the estimated HRs from generalized additive Cox proportional models with the smoothed function of the BMI (in this analysis missing data points were not imputed and model 1 includes similar covariates as the main text).*

|  | ***Cox Propositional Models***  *With BMI in the*  *Model 1 + BMI and Waist C.*  *HR (95% CI)* | ***Generalized Additive Cox Propositional Models***  *With s(BMI) in the*  *Model 1 + BMI and Waist C.*  *HR (95% CI)* |
| --- | --- | --- |
| *IMAT Index* | *1.23 (1.09 - 1.38)* | *1.25 (1.18 – 1.32)* |
| *SAT Index* | *1.33 (1.07 - 1.67)* | *1.31 (1.13 – 1.52)* |
| *PM Density* | *0.93 (0.73 - 1.19)* | *1.00 (0.88 – 1.15)* |

*Considering little effects of non-linearity of BMI in the reported HRs for adipose tissue biomarkers, we did not include the results of these analyses in the main text. Moreover, the alcohol drinking “status” (categorical variable, current vs non-current) was used in this study which limits the opportunity to assess the linearity of the association between alcohol drinking and diabetes incidence.*

**Action**

*N/A*

Results:

18. The authors should document the increase of standard errors or confidence intervals after adjusting BMI and waist circumference. Table 2 clarifies the collinearity of the SAT with BMI and waist circumference.

**Answer**

*Thanks for the comment.*

*The authors acknowledge the concerns of the reviewer. We checked our models for collinearity between SAT and BMI (model 1) by estimating the variance inflation factor (VIF) in the model (in this analysis missing data points were not imputed and model 1 includes similar covariates as the main text):*

|  | ***SAT Index*** | ***Body Mass Index*** | ***Waist Circumference*** |
| --- | --- | --- | --- |
| ***Variance Inflation Factor (VIF)*** | *3.51* | *5.51* | *4.32* |

*Assuming the threshold of 10 for VIF for detecting the multicollinearity (based on the Hair, J. F. Jr., Anderson, R. E., Tatham, R. L. & Black, W. C. (1995). Multivariate Data Analysis (3rd ed). New York: Macmillan), although there was an intermediate level of multicollinearity, this level is acceptable and the estimates from the model are valid.*

*We would like to also mention that after adjusting the statistical models for the BMI and waist circumference, the estimated HRs were attenuated toward the null hypothesis and the estimated confidence intervals were widened. We tried to attenuate the tone of language in the results while discussing results of the models adjusted for BMI and waist circumference, in order to address the concerns raised in this comment.*

**Action**

*Results – Line 249 and 276*

*We tried to attenuate the tone of language in the results while discussing results of the models adjusted for BMI and waist circumference.*

19. Delete S5 Fig or delete S2 Table and include the numeric info into S5 Fig.

**Answer**

*We added the estimated correlation coefficients to the S5 Fig and removed the S2 Table, as requested.*

**Action**

*S5 Fig*

*The estimated correlation coefficients are added to the S5 Fig and the S2 Table is removed, as requested.*

20. Line 228-229. There was no apparent cubic association. Also, the authors did not formally test whether or not there was a cubic or non-linear association. The inflexion point the authors described did not reflect an objective assessment. The authors should delete the sentence without any statistical approach to identify such a value. Also, log-2 values are hard to interpret. The authors should convert it to a raw value to facilitate an interpretation when needed.

**Answer**

*Per reviewers’ comments, we have made several changes to our approach in statistical analysis, including reporting the association between adipose tissue biomarkers and diabetes incidence per 1-SD increment in the biomarkers instead of log2-transformed biomarkers. Following these changes, the generalized additive Cox proportional hazard modeling no longer supports/shows a non-linear association between adipose tissue biomarkers and diabetes incidence. Therefore, the paragraph about this finding is now updated in the results.*

*Moreover, the association between adipose tissue biomarkers and diabetes incidence is now reported per 1-SD increment in the biomarkers instead of log2-transformed analysis, as requested.*

**Action**

*Methods and Results – Throughout*

*Results – Intermuscular Adiposity and T2D Incidence – Line 245*

*The sentence about the non-linear association between IMAT index and diabetes incidence is updated as:*

*“The generalized additive Cox proportional hazard models showed little evidence for a nonlinear association between the IMAT index and T2D incidence (S6 Fig).”*

*Moreover, the association between adipose tissue biomarkers and diabetes incidence is now reported per 1-SD increment in the biomarkers and relevant language is updated accordingly.*

21. The authors should describe the results accounting for the interaction by BMI. It is critically important to state the results after the stratification and after adjusting BMI and waist circumference.

**Answer**

*Please see our answer to comment 0.*

*As explained in the response to comment 0, including the BMI and waist circumference in the models after stratification has little effect on the estimated HRs. As thus, the results of this additional analysis are not included in the main text. Moreover, following the requested changes in the statistical analyses by the editor and reviewers, our models no longer support interaction between BMI and IMAT index in the association with diabetes incidence.*

**Action**

*N/A*

Discussion.

22. The first paragraph is misleading. The association was present among those with overweight and obesity. This should be the basis to characterize the association.

**Answer**

*Thanks for the comment.*

*Per reviewers’ comments, we have made several changes to our approach in statistical analysis, including 1. Multiply imputing the missing data points before Cox proportional hazard modeling, 2. Reporting the association between adipose tissue biomarkers and diabetes incidence per 1-SD increment in the biomarkers (instead of log2-transformed analysis), 3. Correcting the reported p-values for multiple comparisons, and 4. Changing the strata in the BMI-stratified analyses from BMI ≤24.9 vs >24.9 to BMI ≤25.0 vs >25 kg/m2.*

*Following these changes, the reported p-value for the interaction between BMI and IMAT index (in the stratification analysis) is no longer “significant”. Although we have updated and attenuated the language in the abstract, author summary, and discussion to reflect the reviewer’s concerns, we tried not to discuss the “insignificant” p-value for interaction between BMI and IMAT index.*

**Action**

*N/A*

23. The authors' discussion is appropriate for the potential use of CT scanning for the prediction of T2D and other diseases. However, this study is too far from the argument because the authors did not interpret the results quantitatively for the clinical application. Also, this study identified the interaction by BMI and therefore has not granted the wide application of the CT scanning to a general population.

**Answer**

*The authors acknowledge the concerns of the reviewer. We reviewed the discussion, attenuated the tone in this part, and tried to remove the language that implied “establishing the importance of the CT-derived measures to predict T2D risk”. Specifically, we removed all the keywords that implied causality (e.g., risk and predict) and also focused on the pathological pathway behind the association between IMAT and diabetes incidence.*

*However, we kept the few sentences that showed the CT exams we used for our study were primarily performed for other initial indications and also how our finding may extend the value of performing CT exams recommended in clinical guidelines.*

**Action**

*Discussion – Throughout*

*The text has been reviewed and updated as requested.*

The reviewer is reserving other minor comments.

***Reviewer #2 - Statistical review***

This paper reports an observational study that assessed association between biomarkers and risk of developing diabetes. I had some comments on the statistical methods and reporting, which are provided below.

1. Abstract: I'd recommend providing the estimated HR and 95% CI for adjusted SAT association.

**Answer**

*The requested information has been added to the abstract.*

**Action**

*Abstract – Methods and Findings – Line 56*

*The requested information has been added to the abstract. This part reads as:*

*“The IMAT index (HR: 1.27 [95% CI: 1.15-1.41] per 1-standard deviation [SD] increment) and the SAT index (HR: 1.43 [95% CI: 1.16-1.77] per 1-SD increment) at baseline were associated with T2D incidence over the follow-up. The associations of the IMAT and SAT indices with T2D incidence were attenuated after adjustment for body mass index and waist circumference, with HRs of 1.23 (95% CI: 1.09-1.38) and 1.29 (95% CI: 0.96-1.74) per 1-SD increment, respectively.”*

2. Abstract line 62 "can predict a" - I recommend slightly less causal language such as 'is associated with'.

**Answer**

*The language of the conclusion of the abstract has been attenuated and updated as requested and the causal language has been removed.*

**Action**

*Abstract – Conclusions – Line 64*

*The language of the abstract has been attenuated and updated as requested. This part reads as:*

*“In this study, we observed an association between intermuscular adipose tissue at baseline and T2D incidence over the follow-up. This study suggests the potential role of intermuscular adipose depots in the pathophysiology of T2D.”*

3. Abstract/Methods/Results: Were IMAT and SAT included in the same adjusted model? It would be useful to note whether IMAT was an independent risk variable after adjustment for SAT.

**Answer**

*Thanks for the comment.*

*The table below summarizes results of the models when both IMAT and SAT indices are included in the models at the same time (missing data points were multiply imputed and model 1 includes similar covariates as the main text).*

|  |  | ***HR (95% CI), p-value**** |
| --- | --- | --- |
| *Model 0* | *IMAT Index* | *1.24 (1.13 - 1.36), <0.001* |
|  | *SAT Index* | *1.11 (0.95 - 1.29), 0.206* |
| *Model 1* | *IMAT Index* | *1.22 (1.07 - 1.38), 0.003* |
|  | *SAT Index* | *1.18 (0.93 - 1.48), 0.169* |
| *Model 1 + HOMA-IR* | *IMAT Index* | *1.23 (1.08 - 1.40), 0.002* |
|  | *SAT Index* | *1.09 (0.86 - 1.39), 0.459* |
| *Model 1 + BMI and Waist C.* | *IMAT Index* | *1.21 (1.06 - 1.38), 0.004* |
|  | *SAT Index* | *1.09 (0.82 - 1.44), 0.572* |

** p-values are not corrected*

*As shown in the table, the associations between the IMAT index with diabetes incidence seem to be independent of SAT index, and including these two biomarkers in the models at the same time seems to have little effect on the reported results. Therefore, we did not include these results in the main text.*

**Action**

*N/A*

4. Line 197 - I'd recommend adding how many patients were excluded from the complete cases analysis. I think Figure S4 requires more information in the caption as it wasn't clear to me how to interpret it currently.

**Answer**

*Following the reviewer’s requests, the missing data points are now imputed using the Multivariate Imputation by Chained Equations (MICE) and the predictive mean matching method. Moreover, the legend of the S4 Figure is now updated to include more information about the frequency of missing data points in the dataset.*

**Action**

*S4 Fig*

*The legend of S4 Fig is updated to include the following sentence:*

*“In this figure, the blue bars show number of participants with missing data points in the covariates marked with red circles below each bar.”*

5. Statistical analysis: it would be useful to provide a pre-specified analysis plan or otherwise note which analyses described here were specified prior to the data being available.

**Answer**

*The manuscript proposal of this paper, which included the details of study design and statistical analyses, was approved a priori by the MESA publication and steering committees. Although (per MESA policies) we cannot share the manuscript proposal, we have summarized the planned and operationalized approaches of statistical analyses in the S2 Table.*

**Action**

*Method – Statistical Analysis – Line 183*

*S2 Table*

*The S2 Table has been added to the supporting information to highlight the few additions (e.g., assessing the linearity of the associations between adipose tissue biomarkers and diabetes incidence and multiply imputing the missing data points) or changes (e.g., reporting the associations per 1-standard deviation [1-SD] increment in the biomarkers) that were made to the planned statistical analyses at the time of data analysis or per peer-reviewers request.*

6. Line 188-190: I did not follow what the authors meant by 'consecutive nested … were used to estimate' - is this some type of model building procedure? More details on this would be useful.

**Answer**

*The text has been reviewed and the phrase ‘consecutive nested’ was removed.*

**Action**

*Methods – Statistical Analysis – Line 199*

*The text has been reviewed and updated as:*

*“Moreover, several adjusted Cox proportional hazard models were used to estimate the hazard ratios (HRs) and 95% confidence interval (CI) for the T2D incidence according to adipose tissue biomarkers (per 1-SD increment).”*

7. Line 199 - did the stratified analyses also adjust for the variables in the previous paragraph?

**Answer**

*Yes, the models in the stratified analysis included all covariates in Model 1 (traditional risk factors of diabetes), except for the stratification variables.*

**Action**

*N/A*

8. Line 222 - I'd recommend clarifying the 74 does not include the 17 self-identified participants mentioned on line 160?

**Answer**

*Thanks for the comment.*

*Per the reviewer’s request, we updated the diabetes diagnosis criteria to include those participants with (only) self-reported physician-diagnosed disease in the main analysis. The sensitivity analysis is also updated to include only participants with available data about insulin or oral hypoglycemic agents or FPG ≥126 mg/dL in the follow-up exam. Relevant information about this change is now included in the main text.*

**Action**

*N/A*

9. Line 266 - I would add results from the Intramuscular adiposity section to the abstract as currently it looks a bit selective to report only two of the three main sets of results. I'd also add the results on this to table 2 from the supplementary table.

**Answer**

*Thanks for the comment.*

*Results of intramuscular adiposity (PM density) were not added to the abstract due to word count limits in this section. Although the authors acknowledge the concerns of the reviewer, these results are presented in the main text.*

**Action**

*N/A*

***Reviewer #3***

A SUMMARY OF THE STUDY. This manuscript entitled “Adipose tissue biomarkers and risk of type 2 diabetes incidence in normoglycemic participants: The MESArthritis Ancillary Study” provides a thorough investigation of the importance of CT-derived markers for adipose tissue and lipids for the incidence of type 2 diabetes in 1,727 normoglycemic study participants, with an average follow-up time of approximately 7 years.

At baseline, CT-derived adipose tissue biomarkers and pectoralis muscle lipids were examined, along with other metabolic factors, anthropometric measures and classical clinical factors, to assess the predictive ability of these covariates for the development of type 2 diabetes or pre-diabetes.

The authors used Cox proportional hazards model to study the research question and ~75 individuals developed type 2 diabetes during the follow-up time. The authors also performed ancillary analyses that are of interest for the research question.

REMARKS TO THE AUTHOR. The research question is important and the authors provide interesting data to assess this. A strength of the study is the long follow-up time. Although many study participants are included, only a fraction actually developed events during the study period. I have some general questions regarding study design and some concerns about the statistical modeling discussed below.

Study Design and Results – Comments

1. The authors conclude that intermuscular fat (IMAT), but not subcutaneous fat, has a predictive value for risk of developing T2D also after adjusting for BMI and waist circumference. However, causality is not proven and increased intermuscular fat can just be a marker of other ectopic fat depots including liver fat and NAFLD/NASH which also predict risk of T2D. This should be discussed.

**Answer**

*We have reviewed and rewritten the abstract, author summary, methods, and several parts of the discussion and removed the language that implied causality. Instead, we emphasized the “association” between adipose tissue biomarkers and diabetes incidence, highlighting the fact that this biomarker may be a surrogate measure of other ectopic adipose tissues.*

**Action**

*Main Text – Throughout*

*The language that implied causality between adipose tissue biomarkers and diabetes incidence has been removed. The fact that the biomarkers presented in this study may be surrogate measures of other ectopic adipose tissues is emphasized.*

2. The authors mention in the Methods section that IMAT (extramyocellular lipid content), was measured and from this the authors could calculate the intramyocellular lipid content. However, have the authors constructed Cox models for both extra- and intramyocellular lipid content, respectively? I was not able to identify models for both forms of IMAT.

**Answer**

*Thanks for the comment.*

*The IMAT index was a surrogate measure of the extramyocellular adipose tissue while the PM density was a measure of intramyocellular adipose tissue in our study. The statistical models for the association between these biomarkers and diabetes incidence are presented in the main text.*

*Models adjusted for both IMAT index and PM density at the same time are summarized in the table below (missing data points were multiply imputed and model 1 includes similar covariates as the main text).*

|  | | ***HR (95% CI), p-value*** |
| --- | --- | --- |
| *Model 0* | *IMAT Index* | *1.28 (1.18 - 1.39), <0.001* |
|  | *PM Density* | *0.91 (0.76 - 1.09), 0.305* |
| *Model 1* | *IMAT Index* | *1.28 (1.16 - 1.42), <0.001* |
|  | *PM Density* | *0.87 (0.69 - 1.10), 0.252* |
| *Model 1 + HOMA-IR* | *IMAT Index* | *1.26 (1.14 - 1.41), <0.001* |
|  | *PM Density* | *0.93 (0.73 - 1.18), 0.540* |
| *Model 1 + BMI and Waist C.* | *IMAT Index* | *1.24 (1.10 - 1.40), <0.001* |
|  | *PM Density* | *0.90 (0.70 - 1.18), 0.457* |

** p-values are not corrected*

*As shown in the table, the associations between IMAT index and PM density with diabetes incidence seem to be independent of each other, and including these two biomarkers in the models at the same time seems to have little effect on the reported results. Therefore, we did not include these results in the main text.*

**Action**

*N/A*

3. In the Methods section, the authors state that they measured between 2008 and 2010 baseline values for adipose tissue markers from CT examination, classical clinical characteristics, anthropometric measures and HOMA-IR. The authors also mention that these variables were measured again after 9 to 12 months and that a third measurement was performed at the end of the study period. Does this mean that the authors have serial data or time-updated data for all the mentioned covariates? If so, I would suggest redoing the analyses with time-updated mean values in the Cox models, instead of baseline values.

**Answer**

*Thanks for the comment.*

*We don’t have any of “independent” variables or covariates repeatedly examined and measured over time. Therefore performing time-varying covariate analysis is not feasible in our study.*

*Between baseline (April 2010 to January 2012) to December 2017, participants were contacted by interviewers (via phone) at intervals of 9 to 12 months to inquire about new disease diagnoses and interim hospital admissions. The assessments for demographic and clinical characteristics and plasma sample collection were only performed at baseline and follow-up exams and time-updated measures of these variables were not available in the MESA database.*

*We apologize for the text not being clear in this section and made the necessary edits.*

**Action**

*Methods – T2D Diagnosis – Line 150*

*This section has be rewritten to address the issues raised in this comment.*

4. On line 157: According to authors, there are 17 study participants with self-reported type 2 diabetes, but without information on insulin treatment or oral- antidiabetic treatment. Is it possible that these individuals were treated with lifestyle interventions (i.e., improved nutritionary intake and increased physical activity)?

There is a considerable number of T2D patients treated with life-style interventions, ranging between 15-35%. Also, the authors have performed both a supplementary analysis for individuals who developed pre-diabetes and a sensitivity analyses for those that had self-reported T2D. I would not exclude these from the main analyses, if these 17 patients had received a diagnosis of T2D based on fasting plasma glucose levels.

**Answer**

*Due to lack of relevant information, we couldn’t test if these participants (with the self-reported physician-diagnosed disease without available data on insulin or oral hypoglycemic agents or FPG ≥126 mg/dL in the follow-up exam) were treated with lifestyle interventions or not. However (besides the fraction of participants who died over the follow-up and were not able to complete the follow-up exam), being treated with lifestyle modification seems the most likely explanation.*

*As requested, we updated the diabetes diagnosis criteria in our study to include those participants (with the self-reported physician-diagnosed disease without available data about insulin or oral hypoglycemic agents or FPG ≥126 mg/dL in the follow-up exam). Relevant information about this change is now included in the main text and all analyses are updated accordingly.*

**Action**

*Methods – T2D Diagnosis – Line 159 and 164*

*The diabetes diagnosis criteria have been updated as requested, and besides adding relevant information about this change, all analyses have been updated accordingly.*

5. On line 173: Why did the authors use quantiles for IMAT and SAT? Did they use quantiles for the independent covariates, because they had previously modeled all independent variables as linear covariates and discovered that covariates become insignificant?

The authors have used smoothness function for the Cox model to study the pattern of association for some linear covariates. I would suggest that the authors include restricted cubic splines as a regression modelling strategy, with 4 or 5 evenly spaced knots, for all linear covariates in their regression model. This would result in a superior assessment of the covariates and their nonlinear association with incident T2D.

**Answer**

*Thanks for the comment and the concern.*

*No. As you can see, all the tables that present the results of our analysis for each biomarker also include a column showing the association between the “linear” form of adipose tissue biomarker (per 1-SD increment) and diabetes incidence.*

*The generalized smoothness function used in this analysis showed little evidence for a nonlinear association between adipose tissue biomarkers and diabetes incidence. Therefore, the use of cubic splines for regression modeling is not expected to change the estimated results.*

**Action**

*N/A*

6. On line 216: Do the authors consider the overall incidence of T2D in this study to be generalizable to the general population? It is surprising that only 4.3 % of study participants who were 69 years of age, developed T2D during the follow-up time. I would view this incidence as quite low considering the mean age for this study cohort. The incidence of T2D in this age group is nearly 10% in most western populations. Could there be a reason for a such a low incidence?

**Answer**

*The authors acknowledge the concerns raised in this comment. However, we would like to mention that the participants in our study were normoglycemic at the baseline clinical exam and the reported incidence rate of diabetes in our study (103 / 29.8 person-years ~ 3.5, after updating the diabetes diagnosis criteria) is in the acceptable range for this subset of population.*

*For commenting about the generalizability of the findings of this study other factors should be considered, however, the overall incidence rate of diabetes in our study (and in this age group) is comparable to other normoglycemic population.*

**Action**

*N/A*

7. According to Table 2 and as suggested by the authors, the models indicate that there is not a linear association between IMAT- and SAT levels. Therefore, the authors should include a brief paragraph in the discussion regarding this nonlinear association and why the highest quintiles of IMAT and SAT are statistically significant.

**Answer**

*Per reviewers’ comments, we have made several changes to our statistical analyses, including 1. Multiply imputing the missing data points before Cox proportional hazard modeling, 2. Reporting the associations per 1-SD increment in the biomarkers, and 2. Correcting the reported p-values for multiple comparisons. Following these changes, the generalized additive Cox proportional hazard modeling no longer supports/shows a non-linear association between adipose tissue biomarkers and diabetes incidence. Therefore, the language addressing this finding is now updated in the results.*

**Action**

*Results – Intermuscular Adiposity and T2D Incidence – Line 245*

*The sentence about the non-linear association between IMAT index and diabetes incidence is updated as:*

*“The generalized additive Cox proportional hazard models showed little evidence for a nonlinear association between the IMAT index and T2D incidence (S6 Fig).”*

8. This study has not presented data on comorbidities for study participants. Does the cohort gather information on baseline comorbidities, if so, these should be adjusted for along with other cardiometabolic treatments, such as antihypertensive and statins, since these covariates could have modifying effect on the risk of developing T2D?

**Answer**

*Thanks for the comment.*

*We ran our statistical models after including “use of (any) antihypertensive” and “use of statins” as additional covariates in the models. The table below summarizes the results of this additional analysis (missing data points were not multiply imputed and model 1 includes similar covariates as the main text) and compares them with results already presented in the main text.*

| ***Biomarker*** | ***Model*** | | *HR (95% CI)* |
| --- | --- | --- | --- |
| *IMAT Index* | *Model 1 + BMI and Waist C.* | *+ Use of Antihypertensive and Statins* | *1.23 (1.10 – 1.38)* |
|  |  | *-* | *1.23 (1.09 – 1.38)* |
| *SAT Index* | *Model 1 + BMI and Waist C.* | *+ Use of Antihypertensive and Statins* | *1.31 (0.96 – 1.78)* |
|  |  | *-* | *1.29 (0.96 – 1.73)* |
| *PM Density* | *Model 1 + BMI and Waist C.* | *+ Use of Antihypertensive and Statins* | *1.03 (0.79 – 1.33)* |
|  |  | *-* | *0.97 (0.75 – 1.26)* |

*Given the fact the reported results in this table are similar to ones already presented in the main text and the fact that these new variables are not commonly recognized as diabetes risk factors (were not included in our a priori analysis plan), we did not include these results in the main text.*

**Action**

*N/A*

9. Figure 2 should include a box where color for boxes and dots are descripted rather than in legend text. There is a red dot missing in the first stratified model in Figure 2 in the age strata for >60.

**Answer**

*The required information is now added to Fig 2.*

**Action**

*Fig 2*

*Fig 2 is updated as requested.*

10. Why are the models not adjusted for LDL-C? Does the MESA register not include this variable?

**Answer**

*Thanks for the comment.*

*The MESA database does include the LDL cholesterol variable. However, HDL and LDL cholesterols are both representatives of dyslipidemia and previous reports suggest adjusting models for only one of them at a time (especially when the models are also adjusted for triglyceride). For instance, please see the covariates used for adjusting the statistical models in the paper by Flowers et al. (PMID: 30796111) or the paper by Atasoy et al. (PMID: 33865610).*

**Action**

*N/A*

11. From Figure 2, it seems that IMAT interacts with BMI, increasing age and certain ethnicities while SAT interacts with HOMA-IR, sex and ethnicity. It is an interesting aspect that IMAT interacts with older age but high SAT in young individuals was associated with increasing T2D incidence. The authors should discuss this and any possible reasons.

**Answer**

*Thanks for the comment.*

*Per reviewers’ comments, we have made several changes to our approach in statistical analysis, including 1. Multiply imputing the missing data points before Cox proportional hazard modeling, 2. Reporting the association between adipose tissue biomarkers and diabetes incidence per 1-SD increment in the biomarkers (instead of log2-transformed analysis), 3. Correcting the reported p-values for multiple comparisons, and 4. Changing the strata in the BMI-stratified analyses from BMI ≤24.9 vs >24.9 to BMI ≤25.0 vs >25 kg/m2.*

*Following these changes, the reported p-values for the stratified analyses are no longer “statistically significant”. Although we have updated and attenuated the language in the abstract, author summary, and discussion to reflect the reviewer’s concerns, we tried not to discuss the “insignificant” p-values in stratified analyses.*

**Action**

*N/A*

12. In Supplementary Table S6: How do you explain that IMAT and SAT were not predictable of pre-diabetes but of incident T2? Is it simply a case of insufficient events in the statistical models?

**Answer**

*Thanks for the comment.*

*Although a case of insufficient events cannot be ruled out here, the participant with prediabetes at baseline are notified about their diagnosis and are encouraged to engage in daily physical activity and lifestyle modifications and therefore may be subject to “intervention bias”, which could potentially lead to improved body composition (i.e., lower muscular adiposity) and maybe the reason for the lack of association between IMAT and diabetes incidence in this group.*

**Action**

*N/A*

13. Supplementary Figure S4: This figure is difficult to interpret. How was missing data handled in the cox models? Has this something to do with the nested cox-model, which is mentioned in the Methods section? A nested approach to Cox models could mean different things and I assume that it has to do with the nested approach to missing data in the cox proportional hazards model? The authors should have imputed missing data, with either MICE or a machine learning approach, which is considered standard approach for imputing missing values today. If the prevalence of missing values is low, then imputation with mean values could be acceptable. What is the y-axis in Figure S4, proportion or numbers?

**Answer**

*The legend and caption of the y-axis of the S4 Fig are updated to include the requested information (the y-axis in S4 Fig is numbers for each category of missing data points). Moreover, the missing data points in the dataset are multiply imputed using the Multivariate Imputation by Chained Equations (MICE) and the predictive mean matching method, per the reviewer’s request.*

**Action**

*Methods – Statistical Analyses – Line 224*

*S4 Fig*

*The missing data points, before Cox proportional hazard modeling, were multiply imputed. Therefore, the following paragraph is added to the method:*

*“The missing data points were multiply-imputed before Cox proportional hazard modeling and the stratified analyses to produce five datasets, using the Multivariate Imputation by Chained Equations (MICE) and the predictive mean matching method.[27] Each dataset was analyzed separately, and the results were pooled across the datasets using Rubin’s rule (missing values were infrequent among the traditional risk factors of T2D, S4 Fig).”*

*The legend of S4 Fig is also updated as:*

*“In this figure, the blue bars show number of participants with missing data points in the covariates marked with red circles below each bar.”*
