# Supplementary material for: Adipose tissue biomarkers and type 2 diabetes incidence in normoglycemic participants in the MESArthritis Ancillary Study: A cohort study
Source: PLoS Med. 2021 Jul 9;18(7):e1003700. doi: 10.1371/journal.pmed.1003700 (PMC8337053; doi:10.1371/journal.pmed.1003700)
Supplement: Attachment — Submitted filename: Response to reviewers.docx [file pmed.1003700.s018.docx]

Dear editor and reviewers

Thank you so much for reviewing our manuscript. The authors are grateful for the comments. We carefully read the comments and tried to comply with all requests as you can see in the revised version.

To improve the navigation through the letter, we have used color code for the editor as blue (E) and reviewer-1 as green (R1) both in the letter and the annotated version of the main text.

In the end, I would like to thank you for your precious time and scientific comments.  Please do not hesitate to inform us in case of any additional comments.

Sincerely,

Shadpour Demehri MD,

The Russell H. Morgan Department of Radiology and Radiological Science,

Johns Hopkins University School of Medicine,

601 N Caroline St, JHOC 3171c, Baltimore, MD 21287;

Telephone: (410)-416-4179; Fax: (410)-502-6454; Email: Demehri2001@Yahoo.com

***Editors***

**1.** Please remove the word “retrospective” from the title.

**Answer**

*The title of the study is updated as requested.*

**Action**

*Title – Line 2*

*The title of the study is updated to:*

*“Adipose tissue biomarkers and type 2 diabetes incidence in normoglycemic participants in The MESArthritis Ancillary Study: A cohort study”.*

**2.** Please use the "Vancouver" style for reference formatting and see our website for other reference guidelines. For example:

a. Please ensure that journal name abbreviations match those found in the National Center for Biotechnology Information (NCBI) databases, and are appropriately formatted and capitalized. https://journals.plos.org/plosmedicine/s/submission-guidelines#loc-references. For example, in references # 37, 38 etc., the journal titles need to be abbreviated in line with the Vancouver style.

b. Please remove excess text from Reference # 6, “AstraZeneca, MedImmune, Novo Nordisk, and ERX Pharmaceuticals and grants from Aegerion outside the submitted work. Dr Scott is an employee and shareholder in GlaxoSmithKline. Dr Burgess reported grants from Wellcome Trust/Royal Society and the UK Medical Research Council during the conduct of the study. No other disclosures were reported”

**Answer**

*We apologize for the errors. Reference formatting is updated according to the journal guidelines.*

**Action**

*References – Line 373*

*Reference formatting is updated as requested.*

***Reviewer #1***

The authors have revised the manuscript well. Some minor comments are come up with as the followings.

**1.** In the imputation, the authors stated the use of "Multivariate Imputation by Chained Equations (MICE)". The name is correct, according to the publication referred to by the authors. However, the approach has now been well-known as "multiple imputation by chained equations (MICE)". Technically, both are correct, but the latter is standard as far as the Reviewer reviewed some publications. To avoid a potentially negative impression readers may come up with, the authors may want to avoid stating the procedure's name: for example, to state, "imputing missing values with chained equations and predictive mean matching method".

**Answer**

*Text is revised as requested.*

**Action**

*Method – Statistical Analysis – Line 224*

*Text is revised as requested and reads as:*

*“The missing data points were multiply-imputed with chained equations and predictive mean matching method before Cox proportional hazard modeling and the stratified analyses to produce five datasets.”*

**2.** In a couple of tables, p-values presented appeared to be after adjustment for false discovery rate. Some readers would be confused, and therefore the application of the FDR should be noted in the footnote wherever appropriate.

**Answer**

*Footnotes of tables and figures are updated as requested, to highlight the application of the FDR for correcting p-values.*

**Action**

*Tables – Table 2, S3, S4, and S5 Tables*

*Figures – Figure 2, S7, S8, and S9 Figures*

*Footnotes of tables and figures are updated as requested, to highlight the application of the FDR for correcting p-values.*

**3.** S4 Table was not clear what was different from the main table. The authors should confirm that each table and figure stands alone so that readers can understand the material. Also, person-time values across categories should be available. Otherwise, future reviewers cannot use the statistics for meta-analysis readily.

**Answer**

*Footnotes of S4 and S5 tables are updated to present more information about the analyses. Also, the incidence rate of type 2 diabetes (per 1,000 person-years) in the quartiles of adipose tissue biomarkers are added to table 2, S3, S4, and S5 tables.*

**Action**

*Tables – Table 2, S3, S4, and S5 Tables*

*Footnotes of S4 and S5 tables are updated to present more information about the analyses. Also, the incidence rate of type 2 diabetes (per 1,000 person-years) in the quartiles of adipose tissue biomarkers are added to table 2, S3, S4, and S5 tables.*

**4.** In the discussion section, the authors should discuss the possible mechanism of the authors' findings. The current statement is insufficient and very naive (Page 18). The authors should discuss why the IMAT of the chest showed an association with T2D incidence. For example, mechanisms of T2D development include skeletal muscle insulin resistance, hepatic insulin resistance, and beta-cell dysfunction. Then, it is not explicitly clear why the chest IMAT relates to the incidence of T2D. General correlates with IMAT around any tissues are unhelpful.

**Answer**

*The authors would like to thank the reviewer for this comment.*

*We would like to highlight two possible mechanisms for the observed association between intermuscular adipose tissue and type 2 diabetes incidence:*

*(1) Generalized Effect: intermuscular adipose tissue in pectoralis muscles, similar to intermuscular adipose tissues in other skeletal muscles, may play an intermediary role in insulin resistance through secreting pro-inflammatory cytokines, extracellular matrix proteins, and increasing local free fatty acids, and collectively alter the skeletal muscle microenvironment (i.e., the intermuscular adipose tissue in pectoralis muscles is a surrogate measure of the intermuscular adipose tissue in all skeletal muscles).*

*In the paper, we mentioned this mechanism as the main mechanism for the observed association between intermuscular adipose tissue and type 2 diabetes incidence.*

*(2) Localized Effect: intermuscular adipose tissue in pectoralis muscles, as a measure of upper-body intermuscular adipose tissue versus lower-body, may play a specific role in insulin resistance, possibly through a similar mechanism to (or caused by) adipose tissue redistribution in response to secretion of cortisol.*

*Contrary to the former, evidence for the possible localized effect of intermuscular adipose tissue in pectoralis muscles in insulin resistance development (upper- versus lower-body) is limited. To our knowledge, although there are several papers about the differential roles of* ***subcutaneous*** *adipose tissue in the upper and lower bodies in the development of cardiometabolic diseases, currently there is no paper about differential effects of* ***intermuscular*** *adipose tissue in the development of such conditions.*

**Action**

*Discussion – Line 324*

*We discussed the first mechanism in the discussion. However, we tried not to discuss the second mechanism, as in our paper we provided no evidence to support this hypothesis. This section now reads as:*

*“We showed that intermuscular adipose tissue in chest CT exams (i.e., high IMAT index) is associated with T2D incidence. The IMAT index in chest CT exams may reflect the overall (upper and lower bodies) deposition of extramyocellular adipose depots, which is associated with insulin resistance [17, 34, 35] and possibly T2D incidence.”*
